# Supplementary figures and images for: A predictive molecular signature consisting of lncRNAs associated with cellular senescence for the prognosis of lung adenocarcinoma
Source: PLoS One. 2023 Jun 23;18(6):e0287132. doi: 10.1371/journal.pone.0287132 (PMC10289466; doi:10.1371/journal.pone.0287132)

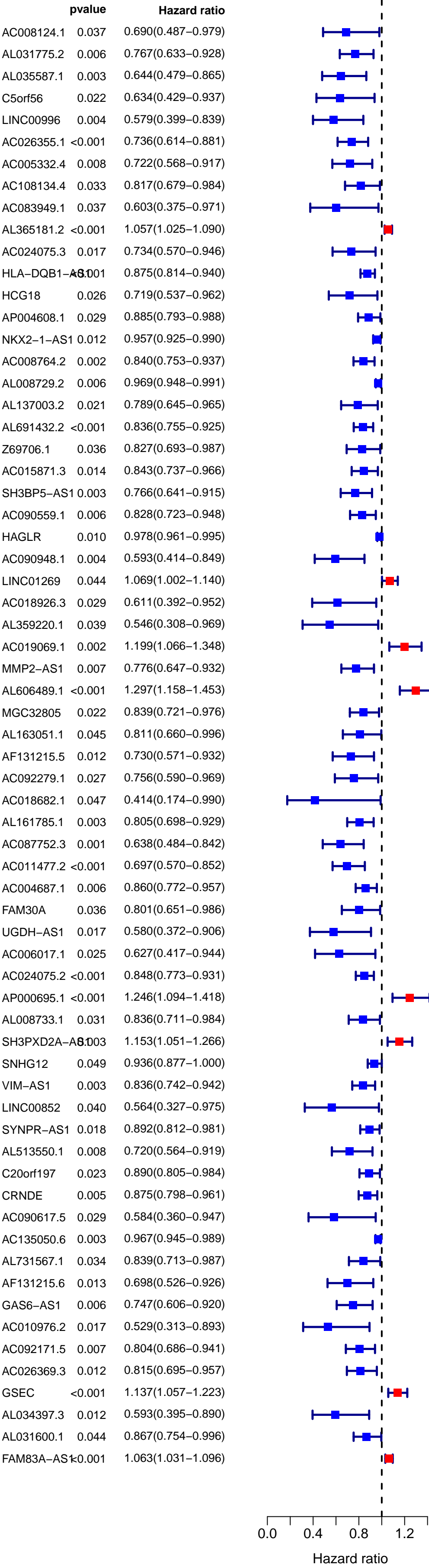

Supplement: S1 Fig — (PDF) [file pone.0287132.s001.pdf]
