# Supplementary material for: A predictive molecular signature consisting of lncRNAs associated with cellular senescence for the prognosis of lung adenocarcinoma
Source: PLoS One. 2023 Jun 23;18(6):e0287132. doi: 10.1371/journal.pone.0287132 (PMC10289466; doi:10.1371/journal.pone.0287132)
Supplement: S1 File — (DOCX) [file pone.0287132.s009.docx]

**S1 File.** 6 lncRNAs recheck and calculations of coding potential

LNCipedia:

AC026355.1

LNCipedia transcript ID: LINC00578:16

LNCipedia gene ID: LINC00578

Ensembl Gene ID: ENSG00000228561

Ensembl Transcript ID: ENST00000423466

Location (hg38): chr3:177683627-177691250

Strand: +

Class: intergenic

Sequence Ontology term: lincRNA

Transcript size: 587 bp

Exons: 4

Sources: NONCODE v4; Ensembl release 75 - Feb 2014; Ensembl release 83 - Dec 2015; Ensembl release 87 - Dec 2016; Ensembl release 90 - Aug 2017; Ensembl release 92 - Apr 2018

Alternative transcript names: NONHSAT093354; ENST00000423466.2

Alternative gene names: ENSG00000228561.2; RP11-114M1.1;LINC00578.

**Coding potential:**PhyloCSF score -273.0829; non-coding

CPAT coding probability 1.05%.non-coding

Reference:

Zhao B, Xu H, Ai X, Adalat Y, Tong Y, Zhang J, et al. Expression profiles of long noncoding RNAs in lung adenocarcinoma. Onco Targets Ther. 2018;11:5383-90. <http://doi.org/10.2147/OTT.S167633>

AL365181.2

LNCipedia transcript ID: lnc-NES-2:3

LNCipedia gene ID: lnc-NES-2

Ensembl Gene ID: ENSG00000272068

Ensembl Transcript ID: ENST00000606343

Location (hg38): chr1:156637783-156641004

Strand: -

Class: bidirectional

Sequence Ontology term:

Transcript size: 3222 bp

Exons: 1

Sources: Ensembl release 75 - Feb 2014; Ensembl release 83 - Dec 2015; Ensembl release 87 - Dec 2016; Ensembl release 90 - Aug 2017; Ensembl release 92 - Apr 2018

Alternative transcript names: ENST00000606343.1

Alternative gene names: ENSG00000272068.1; RP11-284F21.9; **Coding potential:**PhyloCSF score -4.3818; non-coding

CPAT coding probability 10.44%.non-coding

Reference:

Li D, Wang L, Feng J, Shen YW, Liu LN, Wang Y. RP11‑284F21.9 promotes lung carcinoma proliferation and invasion via the regulation of miR‑627‑3p/CCAR1. Oncol Rep. 2020;44(4):1638-48. <http://doi.org/10.3892/or.2020.7732>

Wu G, Wang Q, Zhu T, Fu L, Li Z, Wu Y, et al. Identification and Validation of Immune-Related LncRNA Prognostic Signature for Lung Adenocarcinoma. Front Genet. 2021;12:681277. <http://doi.org/10.3389/fgene.2021.681277>

AF131215.5

LNCipedia transcript ID: lnc-PINX1-84:2

LNCipedia gene ID: lnc-PINX1-84

Ensembl Gene ID: ENSG00000255310

Ensembl Transcript ID: ENST00000530248

Location (hg38): chr8:11107788-11109726

Class: intronic

Sequence Ontology term: sense_intronic_ncRNA

Transcript size: 1939 bp

Exons: 1

Sources: NONCODE v4; Ensembl release 75 - Feb 2014; Ensembl release 83 - Dec 2015; Ensembl release 87 - Dec 2016; Ensembl release 90 - Aug 2017; Ensembl release 92 - Apr 2018

Alternative transcript names: NONHSAT147619; ENST00000530248.2;

Alternative gene names: ENSG00000255310.2; AF131215.2.

**Coding potential:**PhyloCSF score -185.2746; non-coding

CPAT coding probability 3.08%.non-coding

Reference:

Lu Y, Luo X, Wang Q, Chen J, Zhang X, Li Y, et al. A Novel Necroptosis-Related lncRNA Signature Predicts the Prognosis of Lung Adenocarcinoma. Front Genet. 2022;13:862741. <http://doi.org/10.3389/fgene.2022.862741>

C20orf197

LNCipedia transcript ID: lnc-CDH26-9:1

LNCipedia gene ID: lnc-CDH26-9

Ensembl Gene ID: ENSG00000176659

Ensembl Transcript ID: ENST00000625080

Location (hg38): chr20:60055925-60072953

Strand: +

Class: intergenic

Sequence Ontology term: lincRNA

Transcript size: 2732 bp

Exons: 4

Sources: Ensembl release 83 - Dec 2015; Ensembl release 87 - Dec 2016; Ensembl release 90 - Aug 2017; Ensembl release 92 - Apr 2018

Alternative transcript names: ENST00000625080.1

Alternative gene names: ENSG00000176659.7; LINC02910.

**Coding potential:** PhyloCSF score -29.0166; non-coding

CPAT coding probability 30.91%；non-coding

Reference:

Yao J, Chen X, Liu X, Li R, Zhou X, Qu Y. Characterization of a ferroptosis and iron-metabolism related lncRNA signature in lung adenocarcinoma. Cancer Cell Int. 2021;21(1):340. <http://doi.org/10.1186/s12935-021-02027-2>

GAS6-AS1

LNCipedia transcript ID: GAS6-AS1:4

LNCipedia gene ID: GAS6-AS1

Location (hg38): chr13:113842320-113845568

Strand: +

Class: antisense

Sequence Ontology term: antisense_lncRNA

Transcript size: 3249 bp

Exons: 1

Sources: NONCODE v4

Alternative transcript names: NONHSAT035381

Alternative gene names:-

**Coding potential:**PhyloCSF score 6.751; non-coding

LNCipedia transcript ID: GAS6-AS1:5

LNCipedia gene ID: GAS6-AS1

Location (hg38): chr13:113842320-113845746

Strand: +

Class: antisense

Sequence Ontology term: antisense_lncRNA

Transcript size: 3427 bp

Exons: 1

Sources: NONCODE v4

Alternative transcript names: NONHSAT035382

Alternative gene names:-

**Coding potential:**PhyloCSF score 6.751; non-coding

LNCipedia transcript ID: GAS6-AS1:6

HGNC Gene Symbol: GAS6-AS1

HGNC Full Gene Name: GAS6 antisense RNA 1

Ensembl Gene ID: ENSG00000233695

Ensembl Transcript ID: ENST00000611082

Location (hg38): chr13:113842490-113844306

Strand: +

Class: antisense

Sequence Ontology term: antisense_lncRNA

Transcript size: 1817 bp

Exons: 1

Sources: Ensembl release 83 - Dec 2015; Ensembl release 87 - Dec 2016; Ensembl release 90 - Aug 2017; Ensembl release 92 - Apr 2018

Alternative transcript names: ENST00000611082.1

Alternative gene names: ENSG00000233695.2.

**Coding potential:**PhyloCSF score -26.8866; non-coding

Reference:

Han L, Kong R, Yin DD, Zhang EB, Xu TP, De W, et al. Low expression of long noncoding RNA GAS6-AS1 predicts a poor prognosis in patients with NSCLC. Med Oncol. 2013;30(4):694. <http://doi.org/10.1007/s12032-013-0694-5>

GSEC

LNCipedia transcript ID: GSEC:10

LNCipedia gene ID: GSEC

Location (hg38): chr11:126341717-126355587

Strand: -

Class: antisense

Sequence Ontology term: antisense_lncRNA

Transcript size: 752 bp

Exons: 2

Sources: Refseq - Dec 2014

Alternative transcript names: NR_033839

**Coding potential:**PhyloCSF score -93.4002; non-coding

CPAT coding probability12.94%.non-coding

LNCipedia transcript ID: GSEC:11

HGNC Gene Symbol: GSEC

HGNC Full Gene Name: G-quadruplex forming sequence containing lncRNA

Ensembl Gene ID: ENSG00000280832

Ensembl Transcript ID: ENST00000629441

Location (hg38): chr11:126340889-126355579

Strand: -

Class: antisense

Sequence Ontology term: antisense_lncRNA

Transcript size: 1572 bp

Exons: 2

Sources: Ensembl release 83 - Dec 2015; Ensembl release 87 - Dec 2016; Ensembl release 90 - Aug 2017; Ensembl release 92 - Apr 2018

Alternative transcript names: ENST00000629441.1

Alternative gene names: ENSG00000280832.1; ST3GAL4-AS1;

**Coding potential:**PhyloCSF score -28.1544; non-coding

CPAT coding probability 6.58%；non-coding

LNCipedia transcript ID: GSEC:12

LNCipedia gene ID: GSEC

Location (hg38): chr11:126340958-126355536

Strand: -

Class: antisense

Sequence Ontology term: antisense_lncRNA

Transcript size: 1460 bp

Exons: 2

Sources: Biogazelle lncRNA chip; Ensembl release 64 - Sep 2011

Alternative transcript names: ENST00000501321

Alternative gene names: ENSG00000247445; AP001318.3

**Coding potential:**PhyloCSF score -28.1544; non-coding

CPAT coding probability 6.61%；non-coding

.

LNCipedia transcript ID: GSEC:5

LNCipedia gene ID: GSEC

RefSeq ID: NR_033839

Location (hg38): chr11:126341716-126355587

Strand: -

Class: antisense

Sequence Ontology term: antisense_lncRNA

Transcript size: 753 bp

Exons: 2

Sources: Refseq - NCBI Annotation Release 106

Alternative transcript names: NR_033839.1

Alternative gene names: ST3GAL4-AS1

**Coding potential:**PhyloCSF score -93.4002; non-coding

CPAT coding probability 7.14%；non-coding

Reference:

Jiang X, Yuan Y, Tang L, Wang J, Zhang D, Duan L. Systematic Analysis and Validation of the Prognosis, Immunological Role and Biology Function of the Ferroptosis-Related lncRNA GSEC/miRNA-101-3p/CISD1 Axis in Lung Adenocarcinoma. Front Mol Biosci. 2021;8:793732. <http://doi.org/10.3389/fmolb.2021.793732>
