# Supplementary material for: A predictive molecular signature consisting of lncRNAs associated with cellular senescence for the prognosis of lung adenocarcinoma
Source: PLoS One. 2023 Jun 23;18(6):e0287132. doi: 10.1371/journal.pone.0287132 (PMC10289466; doi:10.1371/journal.pone.0287132)
Supplement: Attachment — Submitted filename: PONE-D-23-00324_R1_reviewer.pdf [file pone.0287132.s011.pdf]

PLOS ONE

A predictive molecular signature consisting of lncRNAs related to cellular senescence for the prognosis of lung adenocarcinoma

--Manuscript Draft--

**Manuscript Number:**	PONE-D-23-00324R1
**Article Type:**	Research Article
**Full Title:**	A predictive molecular signature consisting of lncRNAs related to cellular senescence for the prognosis of lung adenocarcinoma
**Short Title:**	A predictive signature consisting of lncRNAs for the prognosis of lung adenocarcinoma
**Corresponding Author:**	Yun Li<br>The Seventh Affiliated Hospital Sun Yat-sen University<br>Shenzhen, CHINA
**Keywords:**	cellular senescence, lung adenocarcinoma, predictive signature, long noncoding RNA, clinical treatment
**Abstract:**	There are few researches and reports on cellular senescence-related long noncoding RNAs (lncRNAs) in terms of lung adenocarcinoma (LUAD). To explore the prognostic role of lncRNAs in LUAD, 279 cellular senescence-related genes, survival information and clinicopathologic parameters were excavated from the CellAge database and The Cancer Genome Atlas (TCGA) database. Then, we constructed a novel cellular senescence-related lncRNAs predictive signature (CS-RLPS) consisting of 6 lncRNAS (AC026355.1, AL365181.2, AF131215.5, C20orf197, GAS6-AS1, GSEC). According to the median of risk score, 480 samples were divided into high-risk and low-risk groups. Furthermore, the clinicopathological and biological functions, immune characteristics and common drug sensitivity were analyzed between two risk groups. In conclusion, the CS-RLPS can independently forecast the prognosis of LUAD, reveals the potential molecular mechanism of cellular senescence-related lncRNAs, and provides appropriate strategies for the clinical treatment of patients with LUAD.
**Order of Authors:**	Anbang Liu
	Xiaohuai Wang
	Liu Hu
	Dongqing Yan
	Yin Yin
	Hongjie Zheng
	Gengqiu Liu
	Junhang Zhang
	Yun Li
**Response to Reviewers:**	Dear Editor:<br>Thank you for giving us the opportunity to revise our manuscript entitled “A novel cellular senescence-related lncRNAs predictive signature for the prognosis of lung adenocarcinoma”. We appreciate the reviewers’ constructive comments and their enthusiasm for our findings. Our point-to-point response to the issues raised by the referees is included. I hope that you would find out the revision is substantial and the reviewers’ concerns have been adequately addressed and agree that the manuscript is now acceptable for publication in PLOS ONE.<br>Sincerely,<br>Yun Li<br>liyun28@mail.sysu.edu.cn<br>Department of Thoracic Surgery, The Seventh Affiliated Hospital, Sun Yat-sen University, Shenzhen, China<br><br>Response to Editor’s comments<br>1. Please ensure that your manuscript meets PLOS ONE's style requirements,

Powered by Editorial Manager® and ProduXion Manager® from Aries Systems Corporation

including those for file naming.
Authors: Thanks for your reminding. We have rechecked our manuscript and introduced minor changes to comply with the journal's style requirements.

2. Please note that PLOS ONE has specific guidelines on code sharing for submissions in which author-generated code underpins the findings in the manuscript. In these cases, all author-generated code must be made available without restrictions upon publication of the work.
Authors: We are very happy to share our code if necessary.

3. About the financial disclosure.
Authors: The authors received no specific funding for this work.

4. In your Data Availability statement, you have not specified where the minimal data set underlying the results described in your manuscript can be found. PLOS defines a study's minimal data set as the underlying data used to reach the conclusions drawn in the manuscript and any additional data required to replicate the reported study findings in their entirety. All PLOS journals require that the minimal data set be made fully available.
Authors: According to your requirements, we will upload our study's minimal underlying data set as supporting information when we re-submit our revised manuscript.

5. PLOS requires an ORCID iD for the corresponding author in Editorial Manager on papers submitted after December 6th, 2016. Please ensure that you have an ORCID iD and that it is validated in Editorial Manager. To do this, go to 'Update my Information' (in the upper left-hand corner of the main menu), and click on the Fetch/Validate link next to the ORCID field. This will take you to the ORCID site and allow you to create a new iD or authenticate a pre-existing iD in Editorial Manager.
Authors: We have already had an ORCID iD and validated it in Editorial Manager. The ORCID iD of the corresponding author is 0000-0003-0949-6519.

6. About the references.
Authors: We appreciate greatly your important warnings and recheck our references. The 14th reference may involve academic misconduct. The 58th reference have been retracted, so we removed the two references and replaced them with relevant current references. Meanwhile, we added two references （the 16th, 17th）and revised the original contents accordingly.

Response to Reviewers' comments
Reviewer #1:
1. Did the authors review and double check the fidelity of the lncRNAs from the databases such as NONCODE etc? For example, C20orf ids indeed might have been reannotated and they could have been coding chunks by the time the paper came out.
Authors: We have rechecked and ensured the fidelity of the 6 lncRNAs in signature from the LNCipedia database. We found the new annotation for the 6 lncRNAs and searched the relevant literature. For example, the alternative gene name of C20orf197 is lnc-CDH26-9, or LINC02910. The following paper came out also mentions the C20orf197. The others' annotation and relevant paper was shown in our uploaded S1 File.
Yao J, Chen X, Liu X, Li R, Zhou X, Qu Y. Characterization of a ferroptosis and iron-metabolism related lncRNA signature in lung adenocarcinoma. Cancer Cell Int. 2021;21(1):340. http://doi.org/10.1186/s12935-021-02027-2

2. Are there any coding potential calculations that the authors looked into these lncRNAs esp looking them as guides (cis or trans)?
Authors: We analyzed the coding potential of the 6 lncRNAs based on the LNCipedia database. All lncRNAs were determined to have no coding ability. The outcome of coding potential is shown in our uploaded S1 File.

3. The PPI and KEGG is well taken, but it would have been nice had authors shown the lncRNA-protein interactions as well to check regulation.
Authors: We showed the correlation analysis between the 6 lncRNAs and coding RNAs in Fig 3C. Regretfully, the interaction mechanism between lncRNAs and protein is so

complicated that we are difficult to visualize them.

4. The abstract must be rewritten.
Authors: We reviewed the section carefully and recognize the seriousness of the problem. We have rewritten and improved the overall structure and logicality of the abstract. The revised abstract will meet hopefully your requirements.

5. The figures are of not high resolution, may be inserted with HR images.
Authors: Thanks for your precious advice. We have revised and improved the resolution of all the figures.

6. Minor but essential, please correct the following:
L61: hepatocellular carcinoma is CLOSE to the WORST overall survival
L192: 39 days OLD
L198: lncRNAs WERE
L201; was SHOWN
l237: scatter plot (tw different words ?)
L389 and L417: through THE
L431: risk groupS
L449: improve THE prognosis
L461: need (remove s)
Authors: We apologize for the mistakes of our manuscript and have revised the above mistakes one by one. We will be happy to edit the text further, based on helpful comments from the reviewers.

Reviewer #2:
1. The study is good. However, needs improvements in terms of overall English language in sections of Abstract, Methods and Results. Please invest enough time and a revision with main authors to bring out the best of the analysis in the text.
Authors: We apologize for the poor language of our manuscript. We have worked on the manuscript for a long time and carefully revised the content of Abstract, Methods and Result, including grammar, conciseness, readability, and logicality. Deletions and additions are both marked. We hope that the language level has been substantially improved.

2. An alternative title could be: "Predictive molecular signature consisting of lncRNA related to cellular senescence for the prognosis of lung adenocarcinoma ". This would be more appropriate.
Authors: That’s a good idea. Thanks for your considerate suggestion. We think that the alternative title is more in line with the overall content of this manuscript. We decide to replace the original title with the new one. Moreover, we would like ask for your opinion. Whether we need add an “A” at the beginning of the new title. “A predictive molecular signature consisting of lncRNAs related to cellular senescence for the prognosis of lung adenocarcinoma”.

3. Materials and methods section could start with explaining the flow diagram of analysis.
Authors: We have adjusted the writing order of the Materials and methods according to the sequence of the flow diagram. It is very sorry that we did not fully understand your advice. Whether you require us to add an explanation of the flow diagram at the beginning of the Materials and methods section or not? Please let us know if it's necessary and we would like to follow up your suggestion.

4. Results section – Each subtitle in this section should highlight the finding or outcome of analysis rather than the process or type of analysis! Please revise, this is very important to improve the quality of presentation of results and also to reach the quality of journal.
Authors: We appreciate greatly your pointing out the shortcomings and have already recognized the seriousness of the problem. We have carefully revised each subtitle to highlight the finding or outcome of analysis.

5. Figures: Please try to improve the resolution of all the figures. Texts in some of the figures is not readable at all.

Powered by Editorial Manager® and ProduXion Manager® from Aries Systems Corporation

	Authors: We are so sorry to bring poor visual experience to you. We have revised and improved the resolution of all the figures.
**Additional Information:**	
**Question**	**Response**
**Financial Disclosure**<br><br>Enter a financial disclosure statement that describes the sources of funding for the work included in this submission. Review the submission guidelines for detailed requirements. View published research articles from *PLOS ONE* for specific examples.<br><br>This statement is required for submission and **will appear in the published article** if the submission is accepted. Please make sure it is accurate.<br><br>**Unfunded studies**<br>Enter: *The author(s) received no specific funding for this work.*<br><br>**Funded studies**<br>Enter a statement with the following details:<br>• Initials of the authors who received each award<br>• Grant numbers awarded to each author<br>• The full name of each funder<br>• URL of each funder website<br>• Did the sponsors or funders play any role in the study design, data collection and analysis, decision to publish, or preparation of the manuscript?<br>• **NO** - Include this sentence at the end of your statement: *The funders had no role in study design, data collection and analysis, decision to publish, or preparation of the manuscript.*<br>• **YES** - Specify the role(s) played.<br><br>* typeset	Include this sentence at the end of your statement: The funders had no role in study design, data collection and analysis, decision to publish, or preparation of the manuscript
**Competing Interests**<br><br>Use the instructions below to enter a competing interest statement for this submission. On behalf of all authors, disclose any competing interests that could be perceived to bias this work—acknowledging all financial support and any other relevant financial or non-	The authors have declared that no competing interests exist.

financial competing interests.<br><br>This statement is required for submission and **will appear in the published article** if the submission is accepted. Please make sure it is accurate and that any funding sources listed in your Funding Information later in the submission form are also declared in your Financial Disclosure statement.<br><br>View published research articles from *PLOS ONE* for specific examples.<br><br>**NO authors have competing interests**<br><br>Enter: *The authors have declared that no competing interests exist.*<br><br>**Authors with competing interests**<br><br>Enter competing interest details beginning with this statement:<br><br>*I have read the journal's policy and the authors of this manuscript have the following competing interests: [insert competing interests here]*<br><br>* typeset	
**Ethics Statement**<br><br>Enter an ethics statement for this submission. This statement is required if the study involved:<br><br>• Human participants<br>• Human specimens or tissue<br>• Vertebrate animals or cephalopods<br>• Vertebrate embryos or tissues<br>• Field research<br><br>Write "N/A" if the submission does not require an ethics statement.<br><br>General guidance is provided below. Consult the submission guidelines for	N/A

detailed instructions. **Make sure that all information entered here is included in the Methods section of the manuscript.**<br><br>**Format for specific study types**<br><br>**Human Subject Research (involving human participants and/or tissue)**<br>• Give the name of the institutional review board or ethics committee that approved the study<br>• Include the approval number and/or a statement indicating approval of this research<br>• Indicate the form of consent obtained (written/oral) or the reason that consent was not obtained (e.g. the data were analyzed anonymously)<br><br>**Animal Research (involving vertebrate animals, embryos or tissues)**<br>• Provide the name of the Institutional Animal Care and Use Committee (IACUC) or other relevant ethics board that reviewed the study protocol, and indicate whether they approved this research or granted a formal waiver of ethical approval<br>• Include an approval number if one was obtained<br>• If the study involved *non-human primates*, add *additional details* about animal welfare and steps taken to ameliorate suffering<br>• If anesthesia, euthanasia, or any kind of animal sacrifice is part of the study, include briefly which substances and/or methods were applied<br><br>**Field Research**<br><br>Include the following details if this study involves the collection of plant, animal, or other materials from a natural setting:<br>• Field permit number<br>• Name of the institution or relevant body that granted permission	
**Data Availability**<br><br>Authors are required to make all data underlying the findings described fully available, without restriction, and from the time of publication. PLOS allows rare	Yes - all data are fully available without restriction

exceptions to address legal and ethical concerns. See the PLOS Data Policy and FAQ for detailed information.<br><br>A Data Availability Statement describing where the data can be found is required at submission. Your answers to this question constitute the Data Availability Statement and **will be published in the article**, if accepted.<br><br>**Important:** Stating 'data available on request from the author' is not sufficient. If your data are only available upon request, select 'No' for the first question and explain your exceptional situation in the text box.<br><br>Do the authors confirm that all data underlying the findings described in their manuscript are fully available without restriction?	
**Describe where the data may be found in full sentences. If you are copying our sample text, replace any instances of XXX with the appropriate details.**<br><br>• If the data are **held or will be held in a public repository**, include URLs, accession numbers or DOIs. If this information will only be available after acceptance, indicate this by ticking the box below. For example: *All XXX files are available from the XXX database (accession number(s) XXX, XXX.*).<br>• If the data are all contained **within the manuscript and/or Supporting Information files**, enter the following: *All relevant data are within the manuscript and its Supporting Information files.*<br>• If neither of these applies but you are able to provide **details of access elsewhere**, with or without limitations, please do so. For example:<br><br>*Data cannot be shared publicly because of [XXX]. Data are available from the XXX Institutional Data Access / Ethics Committee (contact via XXX) for researchers who meet the criteria for*	The sources of public datasets: TCGA database and CellAge database. The article contains the repository/repositories' names and accession number(s).

access to confidential data.<br><br>*The data underlying the results presented in the study are available from (include the name of the third party and contact information or URL).*<br>• This text is appropriate if the data are owned by a third party and authors do not have permission to share the data.<br><br>* typeset	
Additional data availability information:	Tick here if your circumstances are not covered by the questions above and you need the journal's help to make your data available.

Powered by Editorial Manager® and ProduXion Manager® from Aries Systems Corporation

Clean copy Click here to access/download;Manuscript;Manuscript.docx

A predictive molecular signature consisting of lncRNAs related to cellular senescence for the prognosis of lung adenocarcinoma

Anbang Liu[1¶], Xiaohuai Wang[1¶], Liu Hu[1], Dongqing Yan[1], Yin Yin[1], Hongjie Zheng[1], Gengqiu Liu[1], Junhang Zhang[1*] and Yun Li[1*]

[1]Department of Thoracic Surgery, The Seventh Affiliated Hospital, Sun Yat-sen University, Shenzhen, China

[*]liyun28@mail.sysu.edu.cn (YL)

[*]zhangjh33@mail.sysu.edu.cn (JZ)

[¶] These authors contributed equally to this work

Abstract

There are few researches and reports on cellular senescence-related long noncoding RNAs (lncRNAs) in terms of lung adenocarcinoma (LUAD). To explore the prognostic role of lncRNAs in LUAD, 279 cellular senescence-related genes, survival information and clinicopathologic parameters were excavated from the CellAge database and The Cancer Genome Atlas (TCGA) database. Then, we constructed a novel cellular senescence-related lncRNAs predictive signature (CS-RLPS) consisting of 6 lncRNAS (AC026355.1, AL365181.2, AF131215.5, C20orf197, GAS6-AS1, GSEC). According to the median of risk score, 480 samples were divided into high-risk and low-risk groups. Furthermore, the clinicopathological and biological

functions, immune characteristics and common drug sensitivity were analyzed between two risk groups. In conclusion, the CS-RLPS can independently forecast the prognosis of LUAD, reveals the potential molecular mechanism of cellular senescence-related lncRNAs, and provides appropriate strategies for the clinical treatment of patients with LUAD.

Introduction

Globally, among all cancers, lung cancer ranks first in mortality and second in incidence nowadays [1]. Generally, there are two pathological types named non-small cell lung cancer (NSCLC) and small cell lung cancer. Lung adenocarcinoma (LUAD) is the most prevalent histological subtype of primary NSCLC, comprising roughly 50% [2]. According to the different stages of LUAD, patients need to receive comprehensive treatments including surgery, chemoradiotherapy, targeted therapy and immunotherapy, but the prognosis of advanced LUAD is still poor. Therefore, LUAD remains a serious public health problem that requires continuous attention, and it is indispensable to discover more drug targets, biomarkers and improved factors to lead to a better prognosis of patients.

Cellular senescence is characterized by a permanent cessation of cell proliferation. The occurrence of cellular senescence is associated with many different triggers, such as DNA damage and release of inflammatory cytokines, etc. Increasing evidence shows that many aging-related diseases can be

prevented or delayed, including cancer, by blocking-up the biological process of cellular senescence [3, 4]. A large of researches suggest that cellular senescence profoundly regulates tumor microenvironment by promoting the infiltration of some immune cells, activating many signaling pathways and releasing inflammatory cytokines [5-7]. Xue et al. found that in an in vivo model of hepatocellular carcinoma, restoration of *p53* could induce senescence of liver tumor cells and promote elimination of these cells by the innate immune system [8]. Some studies have found that *eIF3A R803K* mutation promotes resistance to chemotherapy by inducing tumor cell senescence and the higher expression of cellular senescence genes in human hepatocellular carcinoma is close to the worst overall survival (OS) [9, 10]. Furthermore, senescent thyroid cells can induce neighboring non-senescent thyroid cancer cells to invade and proliferate. The xenograft model also shows that senescent thyroid cells and cancer cells co-implanted increased lymphovascular invasion and lymph node metastasis through the CXCL12/CXCR4 axis [11].

Long noncoding RNA (LncRNA) refers to a kind of non-coding RNAs that are longer than 200nt and cannot encode proteins. LncRNA can regulate coding genes through chromatin remodeling, transcriptional control and post-transcriptional processing [12]. LncRNA mainly has four functions. The first function is "signaling function", which means the transcript of lncRNA itself serves as the marker of biological events including allelic specificity, structural specificity and various stimuli. Secondly, it is so-called "molecular

decoy" that lncRNA can bind with proteins and miRNAs, etc. The third is "guiding". LncRNA acts in cis or trans to target the bound effector molecule to the position of specific DNA sequence. Finally, lncRNA acts as a "molecular scaffold" can take part in the assembly of transcriptional regulatory complexes [13]. On the basis of previous researches, it has been discovered that lncRNAs with aberrant expression contribute substantially to the onset and development of numerous diseases, and tumor growth and metastasis are particularly influenced by lncRNAs [14]. Several lncRNAs have the potential to be tumor biomarkers and therapeutic targets for the detection and treatment of cancer [15]. However, there are few researches and reports on cellular senescence-related lncRNAs in terms of LUAD.

Through this research, we constructed a cellular senescence-related lncRNAs predictive signature (CS-RLPS), and evaluated its prognostic value in LUAD patients. Furthermore, we further performed immune-related analyses and sensitivity analyses of common drugs to explore a more reasonable clinical treatment plan.

Materials and Methods

Acquisition of LUAD dataset and cellular senescence-related gene set

The Cancer Genome Atlas (TCGA) database (https://portal.gdc.cancer.gov/) was accessible to download the RNA sequencing (RNA-seq) dataset, survival information, and associated clinicopathologic parameters of LUAD. Meanwhile,

we obtained 279 cellular senescence-related genes from the CellAge database (https://genomics.senescence.info/cells/) (S1 Table).

Enrichment analyses of differentially expressed genes related to cellular senescence

We extracted the expression matrix of genes relevant to cellular senescence from the RNA-seq dataset, and used the “limma” package to draw the volcano plot of differentially expressed genes (DEGs). We considered the false discovery rate (FDR) < 0.05 and | log2 fold change (FC) | > 1 as the criteria of significantly differential expression, and further established a protein-protein interaction (PPI) network about DEGs through the STRING database (https://cn.string-db.org/). Then, we visualized the network by Cytoscape software, and screened out hub genes based on degree values by the application “cytoHubba”. The functions of DEGs were excavated by Gene Ontology (GO) and Kyoto Encyclopedia of Genes and Genomes (KEGG) analyses using the R package “clusterProfiler” and “GOplot” [16, 17].

Construction, evaluation and verification of the predictive signature

We calculated the correlation between DEGs and lncRNAs by the “limma” package and regarded the correlation coefficient | R | > 0.3 and p < 0.001 as screening standard. In order to investigate the connection between cellular senescence-related lncRNAs and the OS, the univariate Cox regression analysis was initially performed. Least absolute shrinkage and selection operator

(LASSO) was further used to obtain the cellular senescence-related lncRNAs through the "glmnet" package and taking 10-fold cross-validation [18, 19]. Lastly, we used the multivariate Cox regression analysis to identify and construct an efficient CS-RLPS on the basis of the following computational formula:

$$\text{Risk score} = \sum_{i=1}^{n} \text{Coef(i)} \times \text{Expr(i)}$$

Coef(i) and Expr(i) represent respectively the multivariate Cox regression coefficient and corresponding lncRNA expression level. We could calculate the risk score for each LUAD patient using the formula. Furthermore, on the basis of the median of risk score, we divided LUAD patients into two categories and named respectively high-risk and low-risk groups.

We tested the reliability of the CS-RLPS by principal component analysis (PCA). Additionally, through the "rms" package, we made a nomogram for the purpose of predicting LUAD survival at 1-year, 3-year, and 5-year. The performance of the nomogram was evaluated using calibration curve.

Gene set enrichment analysis (GSEA) 4.1.0 could help to investigate which pathways were mostly enriched in high-risk and low-risk groups, respectively [20]. Normalized enrichment score $|NES| \geq 1$ and nominal $p < 0.05$ were taken into account as our standard for statistical significance.

Immune-related analyses of the CS-RLPS

Immune-related analysis was performed for the CS-RLPS, including the infiltration of stromal and immune cell. We calculated the StromalScore, ImmuneScore, and

ESTIMATEScore respectively by the R package “estimate” and the Wilcoxon signed-rank test was applied to compare immune scores between the different risk groups [21]. Following that, single-sample gene set enrichment analysis (ssGSEA) was used to compute the infiltration scores of immune cells and explore the biological functions of immune-related pathways between high- and low-risk groups through the “GSVA” and “GSEABase” packages [22].

Sensitivity analysis of common drugs

To explore whether the CS-RLPS was a guidance for LUAD therapy, we chose the half-maximal inhibitory concentration (IC50) as a reference to evaluate the predictive role of the signature in chemotherapy and targeted therapy. IC50 of some common drugs for chemotherapy and targeted therapy of LUAD were calculated by the R package “pRRophetic” [23].

Statistical analysis

R software (Version 4.0.5) was carried out conducting all statistical analyses. If not otherwise stated, $p < 0.05$ was thought to be statistically meaningful. The Wilcoxon signed-rank test was utilized to compare differences between two groups. The Kaplan–Meier method was employed for survival analyses by the R package “survminer”and “survival”. We used the “timeROC” and “survivalROC” package to generate the time-dependent and conventional ROC curves. According to the area under the curves (AUCs), the predictive efficacy could be determined.

Results

Identification and analyses of the cellular

senescence-related DEGs

Fig 1 depicts a flow diagram of our work. We obtained 62 cellular senescence-related DEGs, comprising 39 upregulated and 23 downregulated genes (Fig 2A, S2 Table). According to the visual PPI network, we discovered the five most valuable genes as the hub genes based on their degree values (Fig 2B). As was shown in KEGG analyses, the enrichment of DEGs was most prominent along these pathways, such as cellular senescence, cell cycle, the p53 signaling pathway, oocyte meiosis, bladder cancer, cushing syndrome, endocrine resistance and progesterone–mediated oocyte maturation (Fig 2C). Then, GO analyses revealed that these DEGs involved in the cell aging, cellular response to chemical stress, the replicative senescence, negative regulation of mitotic cell cycle, regulation of gliogenesis and so on (Fig 2D).

Fig 1. The flow diagram of our research.

Fig 2. Enrichment analyses. (**A**) The volcano plot of 62 cellular senescence-related DEGs. (**B**) The PPI network of cellular senescence-related DEGs, red squares represent top 5 hub genes. (**C**, **D**) KEGG and GO analysis of cellular senescence-related DEGs, respectively.

Construction and internal validation of the CS-RLPS

We screened out 1081 cellular senescence-related lncRNAs (S3 Table). Moreover, the 480 LUAD patients including both RNA expression data and survival data were

acquired from TCGA dataset, and the OS of these samples was more than 30 days old (S4 Table). 66 lncRNAs were connected to the prognosis of LUAD following the outcomes of the univariate Cox regression analysis **(**S1 Fig, S5 Table**)**. Then, LASSO identified that 29 lncRNAs were involved in cellular senescence and the multivariate Cox regression analysis identified 6 cellular senescence-related lncRNAs (*AC026355.1*, *AL365181.2*, *AF131215.5*, *C20orf197*, *GAS6-AS1*, *GSEC*) **(**S6 and S7 Tables, S1 File**)**. Finally, we constructed the CS-RLPS according to the above analysis. The expression levels of 6 cellular senescence-related lncRNAs were displayed as a heatmap (Fig 3A). Fig 3B showed the correlation between 6 cellular senescence-related lncRNAs and 12 co-expressed mRNAs. The co-expression network of 15 pairs of lncRNA-mRNA was shown in Fig 3C ($| R | > 0.3$ and $p < 0.001$). Among these pairs, *GSEC* was found to be co-expressed with 5 cellular senescence-related genes (*IGFBP3*, *CHEK1*, *G6PD*, *GAPDH* and *ASPH*). *AF131215.5* had co-expressed relationship with 4 cellular senescence-related genes (*ETS1*, *NTN4*, *PDZD2* and *SLC16A7*). *GAS6-AS1* had co-expressed relationship with *CBX7* and *SLC16A7*. *AL513550.1* had co-expressed relationship with *KDM5B* and *SLC16A7*. *AL365181.2* was co-expressed with *G6PD*. *C20orf197* was co-expressed with *WNT16*. We also found that *AL365181.2* and *GSEC* were risk factors, however, the other four lncRNAs were protective factors (*AC026355.1*, *AF131215.5*, *C20orf197*, *GAS6-AS1*) (Fig 3D). We designed a formula used for risk score. Risk score = (-0.270×*AC026355.1* expression, 0.231×*AL365181.2* expression, -0.616×*AF131215.5* expression, -0.580×*C20orf197* expression, -0.627×*GAS6-AS1*

expression, 0.505×*GSEC* expression).

Fig3. Construction of the CS-RLPS. (**A**) The heatmap of 6 cellular senescence-related lncRNAs in tumor and normal tissues. (**B**) The correlation analysis between 6 cellular senescence-related lncRNAs and 12 co-expressed mRNAs. (**C**) The co-expression network of lncRNAs and mRNAs. (**D**) The Sankey diagram for cellular senescence-related lncRNAs, mRNAs and risk status. T, tumor; N, normal.

Correlation between the CS-RLPS and LUAD prognosis

We obtained the median after determining the risk score for each sample using the formula. Subsequently, all samples were divided into high- and low-risk groups based on the median of risk score. The risk scores of two groups were shown in Fig 4A. As the scores climbed, so did the likelihood of death (Fig 4B). Compared with those at high risk, the OS of low-risk patients was noticeably longer (Fig 4C, p=9.925e−10). The AUCs of 1-year, 3-year, and 5-year survival were respectively 0.745, 0.676 and 0.72, which manifested that the signature had relatively good predictive performance (Fig 4F). In order to verify the applicability of CS-RLPS, afterwards, two internal validation cohorts were formed by randomly dividing the LUAD patients. The results of the OS were consistent with the total cohort of predictive signature (Fig 4D, p=1.042e−05; Fig 4E, p=3.293e−05). Meanwhile, the two validation cohorts both showed similar predictive performance to the total cohort according to the ROC curves. The AUCs

of 1-year, 3-year and 5-year survival were respectively 0.728, 0.671 and 0.72 in the first cohort (Fig 4G) and those of the second cohort were respectively 0.762, 0.675 and 0.719 (Fig 4H). PCA 3D plots were used to distribute LUAD patients based on the entire genome, gene sets associated with cellular senescence, cellular senescence-related lncRNAs, and the CS-RLPS (Figs 5A-D). The result identified that the CS-RLPS obviously discriminated risk degree in different quadrants. On the contrary, the other groups could not be effectively distinguished, further supporting the reliability of the CS-RLPS.

Fig4. Correlation between the CS-RLPS and LUAD prognosis. (**A**) The distribution of LUAD patients. (**B**) A scatter plot based on each sample's OS and risk score. (**C**-**E**) The survival curves of LUAD patients in the total cohort and two internal cohorts, respectively. (**F**-**H**) The ROC curves for the total cohort and the two internal cohorts at 1-year, 3-year, and 5-year, respectively.

Fig5. PCA 3D plots based on (**A**) the entire genome, (**B**) the gene sets associated with cellular senescence, (**C**) the cellular senescence-related lncRNAs, (**D**) the CS-RLPS.

Relationship between the CS-RLPS and clinicopathological factors

The univariate Cox regression analysis revealed that stage, T, N and the CS-RLPS were closely connected to the OS of LUAD (Fig 6A). Stage and the CS-RLPS were identified as

two independent predictors after multivariate Cox regression analysis (Fig 6B). Fig 6C indicated that the best predictor among all factors was the risk score in LUAD because of the largest AUC（AUC=0.687）. The differences of all clinicopathological factors were further studied, and the result revealed that N, T, stage and survival state were statistically different from the other factors between the two risk groups (Fig 6D). Next, we obtained the data of 328 LUAD patients including all expression data, survival data and clinicopathological information to investigate the relationship between the CS-RLPS and patients' prognosis. These samples were divided into different clinical subgroups on the basis of different clinicopathological factors. The results indicated that CS-RLPS as a risk factor was a relatively better predictor for LUAD in different clinicopathological subgroups except M1 subgroup (Fig 6E).

Fig6. Relationship between the CS-RLPS and clinicopathological factors. Forest plots for (**A**, **B**) The univariate and multivariate Cox regression analyses of the CS-RLPS and clinicopathological factors. (**C**) The ROC curve of the CS-RLPS and clinicopathological factors. (**D**) Distribution heatmap of 6 lncRNAs and clinicopathological factors. (**E**) A forest plot for clinicopathological subgroup analysis. T, tumor; N, lymph node; M, metastasis. *$p < 0.05$; **$p < 0.01$; ***$p < 0.001$.

The nomogram predicted the prognosis of LUAD

We further constructed a nomogram including stage and the CS-RLPS, which was able to

forecast LUAD survival for 1-year, 3-year, and 5-year (Fig 7A). For the sake of evaluating the predictive efficacy of the signature, the calibration curves were performed and showed that the predictive survival rates were fairly consistent with the actual survival rates at 1-year, 3-year, and 5-year (Figs 7B–D).

Fig7. Nomogram and Calibration. (**A**) A nomogram of predicting LUAD survival at 1-year, 3-year, and 5-year. (**B-D**) The calibration curves for predictive accuracy of the CS-RLPS.

Functional enrichment analysis of the CS-RLPS

Enrichment analysis of the CS-RLPS was further carried out by GSEA to probe the underlying functions between two risk groups (Fig 8). The results indicated that the high-risk patients were connected to tumor-associated pathways, such as DNA replication (NES =2.03 and nominal p <0.001), RNA degradation (NES = 1.81 and nominal p = 0.008), p53 signaling pathway (NES = 1.69 and nominal p = 0.013) and so on. The results also revealed that the low-risk group was closely associated with tumor-immune related pathways, such as FC epsilon RI signaling pathway (NES = -1.86 and nominal p = 0.004), mTOR signaling pathway (NES = -1.63 and nominal p =0.006) and JAK/STAT signaling pathway (NES = -1.62 and nominal p = 0.031). The above results fully proved that cellular senescence-related lncRNAs were linked to tumor immune microenvironment (TIME) in our predictive signature.

Fig8. The signaling pathways of significant enrichment according to GSEA.

Relationship between the CS-RLPS and immune characteristics

The three violin plots respectively showed StromalScore, ImmuneScore and ESTIMATEScore in two risk groups. Notably, the three types of scores were all significantly higher in low-risk patients (Figs 9A–C). To further investigate the association between the CS-RLPS and immune functions, we assessed the ssGSEA enrichment scores for several immune cell subgroups and immune-related pathways. The analysis showed that most of the immune cell infiltration happened in the low-risk group, including T regulatory cells (Tregs), tumor-infiltrating lymphocyte (TIL), T helper cells, neutrophils, mast cells and immature dendritic cells (iDCs) (Fig 9D). There were four immune-related pathways significantly associated with the CS-RLPS, including type II Interferon (IFN) response, T cell co-inhibition, major histocompatibility complex (MHC) class I, and human leukocyte antigen (HLA) (Fig 9E). Furthermore, we discovered that the genes related to immune checkpoints were highly expressed in the low-risk group, particularly, such as *ICOS*, *BTNL2*, *TNFSF14*, *CD160*, *CD80*, *BTLA*, *TNFRSF25*, *TNFRSF14*, *CD27*, *CD200R1*, *CD28*, *CD48*, *TNFSF15*, *CD40LG*, *ADORA2A*, *IDO2* and *CTLA4*, etc. (Fig 9F).

Fig9. Immune-related Analyses. (**A**-**C**) The violin plots of StromalScore, ImmuneScore, and ESTIMATEScore between two groups with high-and low-risk.

(**D**, **E**) The enrichment scores of immune cells and immune-related pathways between two groups with high-and low-risk. (**F**) The expression of immune checkpoint between two groups with high-and low-risk. T, tumor; N, normal; M, metastasis; ns, non-significant; *p < 0.05; **p < 0.01; ***p < 0.001.

Predicting efficacy of the CS-RLPS in LUAD therapy

To explore whether the CS-RLPS was a guidance for LUAD therapy, we obtained the IC50 of some conventional chemotherapy drugs and targeted drugs as pharmacological data. The results showed that the IC50 of cisplatin, docetaxel, etoposide, paclitaxel, gemcitabine, erlotinib, crizotinib and afatinib was lower in high-risk group, meaning that the high-risk individuals were more sensitive to the majority of common chemotherapy drugs and targeted drugs (Figs 10A-H). Therefore, the CS-RLPS could be used for predicting the efficacy of LUAD therapy and provided appropriate clinical guidance.

Fig10. The sensitivity analysis of common chemotherapeutic and targeted drugs. (**A-E**) The chemotherapy drugs including Cisplatin, Docetaxel, Etoposide, Paclitaxel, Gemcitabine. (**F-H**) The targeted drugs including Erlotinib, PF.02341066 (Crizotinib) and BIBW2992 (Afatinib).

Discussion

Currently, LUAD is the most epidemic pathological type that threatens non-smokers,

and its incidence is persistently increasing year by year. Even though there are a host of treatments available, the prognosis of advanced LUAD remains very poor [24]. With the deepening research on tumor suppressor mechanism, the prevention of tumorigenesis and development by inducing tumor cellular senescence is gradually receiving attention. Cellular senescence is an anti-proliferative program that leads to permanent cell growth arrest and protects cells from unnecessary damage [25, 26]. In this study, we constructed a CS-RLPS for predicting the prognosis of LUAD. We obtained 62 cellular senescence-related DEGs, of which 5 genes were most closely associated with the other genes. Among them, *CDKN2A* encodes a nucleolar protein involved in triggering cell cycle arrest and apoptosis to suppress tumor growth [27]. Studies have shown that *CDKN2A* represses *MDM2*, a negative regulator of *p53*, thereby stabilizing *p53* and enabling it to generate a transcriptional response by relocating it to the nucleolus [28]. Liu et al. found that *CDKN2A* loss promoted the progression of lung cancer and was closely associated with poorer survival outcomes [29]. Cyclin dependent kinase 1 (*CDK1*) is one of the most important factors regulating the cell cycle, which is crucial for the time phase transitions of the cell cycle. Multiple bioinformatics analyses suggest that *CDK1* expressed highly in LUAD, negatively correlated to the OS of individuals with LUAD [30-32]. Li et al. showed that *miR-143-3p* could target and regulate *CDK1* and promote cancer cell apoptosis in LUAD A549 cell line [33].

In the enrichment analyses of 62 DEGs, we were pleasantly surprised to find that there was a significant enrichment in the *p53* pathway. A great deal of genes

mediating cell cycle arrest can be regulated via *p53* signaling pathway [34]. The pathway is also involved in regulating cellular senescence, which is consistent with our enrichment results [34, 35]. Previous evidence has shown that the *p53* pathway can accelerate apoptosis and autophagy of LUAD cells [36, 37]. Based on enrichment results, we believe that cellular senescence-related genes can inhibit the biological behavior of LUAD through the *p53* pathway.

The role of lncRNAs in LUAD has been verified by more and more researches. However, there are few reports on the role of lncRNAs related to cellular senescence in LUAD patients. Roth et al. indicated that down-regulating the expression of *linc00673* could trigger the senescence of LUAD cells, thereby exerting an inhibitory effect on LUAD [38]. Tao et al. confirmed that lncRNA *MEG3* promoted etoposide-induced tumor cell senescence through the *miR-16-5p*/*VGLL4* axis [39]. In our study, we ultimately identified 6 cellular senescence-related lncRNAs (*AF131215.5*, *GAS6-AS1*, *AC026355.1*, *GSEC*, *AL365181.2*, *C20orf197*) through multivariate COX regression analysis for building the predictive signature. We also found that 12 mRNAs (*ETS1*, *CBX7*, *SLC16A7*, *NTN4*, *PDZD2*, *KDM5B*, *WNT16*, *ASPH*, *IGFBP3*, *CHEK1*, *G6PD*, *GAPDH*) co-expressed with these 6 lncRNAs. Among them, high expression of NTN4 attenuates DNA damage-induced senescence in Glioblastoma multiforme (GBM) [40]. *PDZD2* is able to induce senescence in prostate cancer, breast cancer and liver cancer cells via the *p53* signaling pathway [41]. Knockdown of *GAPDH* can trigger cellular senescence in A549 cell line. Then, further experiments have found that *GAPDH* depletion may

induce and accelerate cellular senescence of tumor through the *AMPK* network in the absence of DNA damage [42]. Besides, high expression of *G6PD* can also promote the growth of glioblastoma and inhibit its senescence [43].

In this research, LUAD patients were categorized as high and low risk according to the median of risk score. The ROC curve suggested that the CS-RLPS had relatively outstanding predictive efficacy. Moreover, we randomly divided these patients into two internal validation cohorts and plotted ROC curves separately. The results suggested that they had the semblable predictive performance as the total cohort. The analysis of clinicopathological factors led to the conclusion that the CS-RLPS could be regarded as an independently prognostic indicator of LUAD patients.

GSEA showed that JAK/STAT pathway, mTOR pathway and FC epsilon RI pathway was enriched notably in the low-risk group. Thereinto, FC epsilon RI signaling pathway has many connections with immune function [44], while mTOR signaling pathway and JAK/STAT signaling pathway are closely associated with cancers. Many researches have indicated that the mTOR signaling pathway is one of the most common regulatory pathways which affected the occurrence, development and metastasis of LUAD by inducing cell apoptosis [45-47]. Rapamycin and temsirolimus as the mTOR inhibitors have already been used to treat NSCLC in various phases of clinical trials [48, 49]. Currently, in accordance with more and more genetic and pharmacological evidence, mTOR activity is recognized as a main driver of cellular senescence [50]. Carroll B et al. found that *mTORC1* hyperactivity was an obvious characteristic of cellular senescence, which could arrest the cell

cycle [51]. Moreover, it was found that senescent cells produced pro-inflammatory cytokines to exacerbate aging-related tissue function decline by utilizing translational programs downstream of *mTORC1* [52, 53]. The research showed that mTOR inhibition could not extend longevity of ATG-deficient worms, suggesting that the process of modulating lifespan through the mTOR signaling pathway might depend on autophagy mechanisms [54]. In addition, the rapamycin was found to extend lifespan in mice, further indicating the stimulative ageing effect of mTOR signaling pathway in the cellular senescence [55]. Based on the above studies, we believe that cellular senescence is a vital biological process in regulating LUAD via the mTOR signaling pathway. Furthermore, the JAK/STAT signaling pathway is also considered to be closely associated with biological behaviors of lung cancer, such as drug resistance [56]. Xu et al. verified that *JAK2* was positively linked to the proliferation and invasion of A549 cells [57]. There are also some evidences indicate that the JAK/STAT signaling pathway in senescent cells is more active than non-senescent cells and inhibition of the pathway can suppress the aging-related secretory phenotype and alleviate dysfunction in senescent cells [58].

The analysis of TIME showed StromalScore, ImmuneScore and ESTIMATEScore were significantly higher in low-risk groups. Therefore, we speculated that antitumor immunotherapy had a good therapeutic effect for the low-risk patients. Subsequently, ssGSEA also revealed a variety of immune cells, as well as some immune-related pathways, had higher immune scores in the group with low risk. Mayoux et al. demonstrated that DCs determined the treatment efficacy of PD-L1 immune

checkpoint inhibitors [59]. A DC gene signature was strongly related to improvement of the OS in NSCLC patients through PD-L1 blockade treatment. Shikotra et al. discovered that the mast cells with different phenotypes were increased in the NSCLC, and the production of TNF-α by both mast cell phenotypes was particularly important for their interaction with other immune cells and the associated inhibition of tumor progression [60]. Researchers found that the Tregs with increased number and enhanced activity in the peripheral blood, metastatic lymph nodes and the tumors were positively related to the recurrence and metastasis of patients with NSCLC [61, 62]. The immune checkpoint inhibitors were received more and more attention in recent years. We further analyzed the expression condition of genes related to immune checkpoint and revealed that the majority of checkpoints were more active in the low-risk group. These outcomes demonstrated that the patients at low risk were probably more responsive to immunotherapy, and the immune checkpoint inhibitors would improve the prognosis of these patients. Among these immune checkpoints, CTLA-4 inhibitors, as the effective drugs, were a milestone in the treatment of NSCLC [63]. Besides, *TIGIT* and *CD27* related immune checkpoints inhibitors have been under investigation in preclinical phase [64]. Lastly, our research showed that it was probably more advantageous for high-risk patients to accept the conventional chemotherapy drugs (cisplatin, docetaxel, etoposide, paclitaxel, and gemcitabine) and targeted therapy (erlotinib, crizotinib and afatinib).

To sum up, most of the results were helpful to explore a more precise and

personalized therapeutic schedule for LUAD patients. Nevertheless, some limitations of our design are inevitable. Firstly, this study still requires an appropriate external dataset to validate the applicability of the CS-RLPS. Secondly, the molecular mechanism of cellular senescence-related lncRNAs in LUAD needs further experimental verification.

Conclusion

In summary, the CS-RLPS can be regarded as an independently prognostic indicator of LUAD patients. It is helpful to explore the underlying molecular mechanism of lncRNAs related to cellular senescence in LUAD. From the perspective of clinical guidance, the CS-RLPS provides relatively scientific treatment strategies for LUAD patients. However, future experiments will be necessary to confirm its validity once more.

Acknowledgments

We express gratitude to contributions from TCGA database and CellAge database.

Author Contributions

Conception and design: AL, XW, YL and JZ.

Data collection: AL, XW and YL.

Statistical analysis and interpretation of data: AL, XW, DY, YY and YL.

Preparation of the figures: AL, XW, HZ, GL and JZ. The first draft writing: AL, XW and LH.

Review and revise of the manuscript: YL and JZ.

References

1. Sung H, Ferlay J, Siegel RL, Laversanne M, Soerjomataram I, Jemal A, et al. Global Cancer Statistics 2020: GLOBOCAN Estimates of Incidence and Mortality Worldwide for 36 Cancers in 185 Countries. CA Cancer J Clin. 2021;71(3):209-49. http://doi.org/10.3322/caac.21660
2. Thai AA, Solomon BJ, Sequist LV, Gainor JF, Heist RS. Lung cancer. Lancet. 2021;398(10299):535-54. http://doi.org/10.1016/S0140-6736(21)00312-3
3. Hernandez-Segura A, Nehme J, Demaria M. Hallmarks of Cellular Senescence. Trends Cell Biol. 2018;28(6):436-53. http://doi.org/10.1016/j.tcb.2018.02.001
4. Demaria M, O'Leary MN, Chang J, Shao L, Liu S, Alimirah F, et al. Cellular Senescence Promotes Adverse Effects of Chemotherapy and Cancer Relapse. Cancer Discov. 2017;7(2):165-76. http://doi.org/10.1158/2159-8290.CD-16-0241
5. Lasry A, Ben-Neriah Y. Senescence-associated inflammatory responses: aging and cancer perspectives. Trends Immunol. 2015;36(4):217-28. http://doi.org/10.1016/j.it.2015.02.009
6. Coppé JP, Desprez PY, Krtolica A, Campisi J. The senescence-associated secretory phenotype: the dark side of tumor suppression. Annu Rev Pathol. 2010;5:99-118. http://doi.org/10.1146/annurev-pathol-121808-102144
7. Lopes-Paciencia S, Saint-Germain E, Rowell MC, Ruiz AF, Kalegari P, Ferbeyre G. The

senescence-associated secretory phenotype and its regulation. Cytokine. 2019;117:15-22. http://doi.org/10.1016/j.cyto.2019.01.013

8. Xue W, Zender L, Miething C, Dickins RA, Hernando E, Krizhanovsky V, et al. Senescence and tumour clearance is triggered by p53 restoration in murine liver carcinomas. Nature. 2007;445(7128):656-60. http://doi.org/10.1038/nature05529

9. Chen YX, Wang CJ, Xiao DS, He BM, Li M, Yi XP, et al. eIF3a R803K mutation mediates chemotherapy resistance by inducing cellular senescence in small cell lung cancer. Pharmacol Res. 2021;174:105934. http://doi.org/10.1016/j.phrs.2021.105934

10. Eggert T, Wolter K, Ji J, Ma C, Yevsa T, Klotz S, et al. Distinct Functions of Senescence-Associated Immune Responses in Liver Tumor Surveillance and Tumor Progression. Cancer Cell. 2016;30(4):533-47. http://doi.org/10.1016/j.ccell.2016.09.003

11. Kim YH, Choi YW, Lee J, Soh EY, Kim JH, Park TJ. Senescent tumor cells lead the collective invasion in thyroid cancer. Nat Commun. 2017;8:15208. http://doi.org/10.1038/ncomms15208

12. Statello L, Guo CJ, Chen LL, Huarte M. Gene regulation by long non-coding RNAs and its biological functions. Nat Rev Mol Cell Biol. 2021;22(2):96-118. http://doi.org/10.1038/s41580-020-00315-9

13. Wang KC, Chang HY. Molecular mechanisms of long noncoding RNAs. Mol Cell. 2011;43(6):904-14. http://doi.org/10.1016/j.molcel.2011.08.018

14. Fernandes JCR, Acuna SM, Aoki JI, Floeter-Winter LM, Muxel SM. Long Non-Coding RNAs in the Regulation of Gene Expression: Physiology and Disease. NON-CODING RNA. 2019;5(171). http://doi.org/10.3390/ncrna5010017

15. Peng F, Wang R, Zhang Y, Zhao Z, Zhou W, Chang Z, et al. Differential expression analysis at the

individual level reveals a lncRNA prognostic signature for lung adenocarcinoma. Mol Cancer. 2017;16(1):98. http://doi.org/10.1186/s12943-017-0666-z

16. Yu G, Wang LG, Han Y, He QY. clusterProfiler: an R package for comparing biological themes among gene clusters. Omics. 2012;16(5):284-7. http://doi.org/10.1089/omi.2011.0118
17. Walter W, Sánchez-Cabo F, Ricote M. GOplot: an R package for visually combining expression data with functional analysis. Bioinformatics. 2015;31(17):2912-4. http://doi.org/10.1093/bioinformatics/btv300
18. Tibshirani R. The lasso method for variable selection in the Cox model. Stat Med. 1997;16(4):385-95. http://doi.org/10.1002/(sici)1097-0258(19970228)16:4<385::aid-sim380>3.0.co;2-3
19. Friedman J, Hastie T, Tibshirani R. Regularization Paths for Generalized Linear Models via Coordinate Descent. J Stat Softw. 2010;33(1):1-22.
20. Subramanian A, Tamayo P, Mootha VK, Mukherjee S, Ebert BL, Gillette MA, et al. Gene set enrichment analysis: a knowledge-based approach for interpreting genome-wide expression profiles. Proc Natl Acad Sci U S A. 2005;102(43):15545-50. http://doi.org/10.1073/pnas.0506580102
21. Yoshihara K, Shahmoradgoli M, Martínez E, Vegesna R, Kim H, Torres-Garcia W, et al. Inferring tumour purity and stromal and immune cell admixture from expression data. Nat Commun. 2013;4:2612. http://doi.org/10.1038/ncomms3612
22. Rooney MS, Shukla SA, Wu CJ, Getz G, Hacohen N. Molecular and genetic properties of tumors associated with local immune cytolytic activity. Cell. 2015;160(1-2):48-61. http://doi.org/10.1016/j.cell.2014.12.033

23. Geeleher P, Cox N, Huang RS. pRRophetic: an R package for prediction of clinical chemotherapeutic response from tumor gene expression levels. Plos One. 2014;9(9):e107468. http://doi.org/10.1371/journal.pone.0107468
24. Allemani C, Matsuda T, Di Carlo V, Harewood R, Matz M, Nikšić M, et al. Global surveillance of trends in cancer survival 2000-14 (CONCORD-3): analysis of individual records for 37 513 025 patients diagnosed with one of 18 cancers from 322 population-based registries in 71 countries. Lancet. 2018;391(10125):1023-75. http://doi.org/10.1016/S0140-6736(17)33326-3
25. Kuilman T, Michaloglou C, Mooi WJ, Peeper DS. The essence of senescence. Genes Dev. 2010;24(22):2463-79. http://doi.org/10.1101/gad.1971610
26. Calcinotto A, Kohli J, Zagato E, Pellegrini L, Demaria M, Alimonti A. Cellular Senescence: Aging, Cancer, and Injury. Physiol Rev. 2019;99(2):1047-78. http://doi.org/10.1152/physrev.00020.2018
27. Weber JD, Taylor LJ, Roussel MF, Sherr CJ, Bar-Sagi D. Nucleolar Arf sequesters Mdm2 and activates p53. Nat Cell Biol. 1999;1(1):20-6. http://doi.org/10.1038/8991
28. Kamijo T, Zindy F, Roussel MF, Quelle DE, Downing JR, Ashmun RA, et al. Tumor suppression at the mouse INK4a locus mediated by the alternative reading frame product p19ARF. Cell. 1997;91(5):649-59. http://doi.org/10.1016/s0092-8674(00)80452-3
29. Liu W, Zhuang C, Huang T, Yang S, Zhang M, Lin B, et al. Loss of CDKN2A at chromosome 9 has a poor clinical prognosis and promotes lung cancer progression. Mol Genet Genomic Med. 2020;8(12):e1521. http://doi.org/10.1002/mgg3.1521
30. Li S, Li H, Cao Y, Geng H, Ren F, Li K, et al. Integrated bioinformatics analysis reveals CDK1 and PLK1 as potential therapeutic targets of lung adenocarcinoma. Medicine (Baltimore). 2021;100(32):e26474. http://doi.org/10.1097/MD.0000000000026474

31. Li M, He F, Zhang Z, Xiang Z, Hu D. CDK1 serves as a potential prognostic biomarker and target for lung cancer. J Int Med Res. 2020;48(2):1219697060. http://doi.org/10.1177/0300060519897508

32. Chen C, Tang Y, Qu WD, Han X, Zuo JB, Cai QY, et al. Evaluation of clinical value and potential mechanism of MTFR2 in lung adenocarcinoma via bioinformatics. Bmc Cancer. 2021;21(1):619. http://doi.org/10.1186/s12885-021-08378-3

33. Li Q, Bian Y, Li Q. Down-Regulation of TMPO-AS1 Induces Apoptosis in Lung Carcinoma Cells by Regulating miR-143-3p/CDK1 Axis. Technol Cancer Res Treat. 2021;20:1079216528. http://doi.org/10.1177/1533033820948880

34. Fischer M. Census and evaluation of p53 target genes. Oncogene. 2017;36(28):3943-56. http://doi.org/10.1038/onc.2016.502

35. Gu Z, Jiang J, Tan W, Xia Y, Cao H, Meng Y, et al. p53/p21 Pathway involved in mediating cellular senescence of bone marrow-derived mesenchymal stem cells from systemic lupus erythematosus patients. Clin Dev Immunol. 2013;2013:134243. http://doi.org/10.1155/2013/134243

36. Li H, Zhang H, Huang G, Bing Z, Xu D, Liu J, et al. Loss of RPS27a expression regulates the cell cycle, apoptosis, and proliferation via the RPL11-MDM2-p53 pathway in lung adenocarcinoma cells. J Exp Clin Cancer Res. 2022;41(1):33. http://doi.org/10.1186/s13046-021-02230-z

37. Shen D, Zeng Y, Zhang W, Li Y, Zhu J, Liu Z, et al. Chenodeoxycholic acid inhibits lung adenocarcinoma progression via the integrin α5β1/FAK/p53 signaling pathway. Eur J Pharmacol. 2022;923:174925. http://doi.org/10.1016/j.ejphar.2022.174925

38. Roth A, Boulay K, Groß M, Polycarpou-Schwarz M, Mallette FA, Regnier M, et al. Targeting LINC00673 expression triggers cellular senescence in lung cancer. Rna Biol. 2018;15(12):1499-511. http://doi.org/10.1080/15476286.2018.1553481

39. Tao Y, Yue P, Miao Y, Gao S, Wang B, Leng SX, et al. The lncRNA MEG3/miR-16-5p/VGLL4 regulatory axis is involved in etoposide-induced senescence of tumor cells. J Gene Med. 2021;23(2):e3291. http://doi.org/10.1002/jgm.3291

40. Li L, Huang Y, Gao Y, Shi T, Xu Y, Li H, et al. EGF/EGFR upregulates and cooperates with Netrin-4 to protect glioblastoma cells from DNA damage-induced senescence. Bmc Cancer. 2018;18(1):1215. http://doi.org/10.1186/s12885-018-5056-4

41. Tam CW, Liu VW, Leung WY, Yao KM, Shiu SY. The autocrine human secreted PDZ domain-containing protein 2 (sPDZD2) induces senescence or quiescence of prostate, breast and liver cancer cells via transcriptional activation of p53. Cancer Lett. 2008;271(1):64-80. http://doi.org/10.1016/j.canlet.2008.05.047

42. Phadke M, Krynetskaia N, Mishra A, Krynetskiy E. Accelerated cellular senescence phenotype of GAPDH-depleted human lung carcinoma cells. Biochem Biophys Res Commun. 2011;411(2):409-15. http://doi.org/10.1016/j.bbrc.2011.06.165

43. Sun M, Sheng H, Wu T, Song J, Sun H, Wang Y, et al. PIKE-A promotes glioblastoma growth by driving PPP flux through increasing G6PD expression mediated by phosphorylation of STAT3. Biochem Pharmacol. 2021;192:114736. http://doi.org/10.1016/j.bcp.2021.114736

44. Li J, Liu R, Sun M, Wang J, Wang N, Zhang X, et al. The FcεRI signaling pathway is involved in the pathogenesis of lacrimal gland benign lymphoepithelial lesions as shown by transcriptomic analysis. Sci Rep. 2021;11(1):21853. http://doi.org/10.1038/s41598-021-01395-z

45. Wang Q, Liu Z, Du K, Liang M, Zhu X, Yu Z, et al. Babaodan inhibits cell growth by inducing autophagy through the PI3K/AKT/mTOR pathway and enhances antitumor effects of cisplatin in NSCLC cells. Am J Transl Res. 2019;11(8):5272-83.

46. Hu T, Shen H, Huang H, Yang Z, Zhou Y, Zhao G. Cholesterol-lowering drug pitavastatin targets lung cancer and angiogenesis via suppressing prenylation-dependent Ras/Raf/MEK and PI3K/Akt/mTOR signaling. Anticancer Drugs. 2020;31(4):377-84. http://doi.org/10.1097/CAD.0000000000000885

47. Chen M, Zhu LL, Su JL, Li GL, Wang J, Zhang YN. Prucalopride inhibits lung cancer cell proliferation, invasion, and migration through blocking of the PI3K/AKT/mTor signaling pathway. Hum Exp Toxicol. 2020;39(2):173-81. http://doi.org/10.1177/0960327119883409

48. Waqar SN, Gopalan PK, Williams K, Devarakonda S, Govindan R. A phase I trial of sunitinib and rapamycin in patients with advanced non-small cell lung cancer. Chemotherapy. 2013;59(1):8-13. http://doi.org/10.1159/000348584

49. Waqar SN, Baggstrom MQ, Morgensztern D, Williams K, Rigden C, Govindan R. A Phase I Trial of Temsirolimus and Pemetrexed in Patients with Advanced Non-Small Cell Lung Cancer. Chemotherapy. 2016;61(3):144-7. http://doi.org/10.1159/000442147

50. Liu GY, Sabatini DM. mTOR at the nexus of nutrition, growth, ageing and disease. Nat Rev Mol Cell Biol. 2020;21(4):183-203. http://doi.org/10.1038/s41580-019-0199-y

51. Carroll B, Nelson G, Rabanal-Ruiz Y, Kucheryavenko O, Dunhill-Turner NA, Chesterman CC, et al. Persistent mTORC1 signaling in cell senescence results from defects in amino acid and growth factor sensing. J Cell Biol. 2017;216(7):1949-57. http://doi.org/10.1083/jcb.201610113

52. Laberge RM, Sun Y, Orjalo AV, Patil CK, Freund A, Zhou L, et al. MTOR regulates the pro-tumorigenic senescence-associated secretory phenotype by promoting IL1A translation. Nat Cell Biol. 2015;17(8):1049-61. http://doi.org/10.1038/ncb3195

53. Herranz N, Gallage S, Mellone M, Wuestefeld T, Klotz S, Hanley CJ, et al. mTOR regulates

MAPKAPK2 translation to control the senescence-associated secretory phenotype. Nat Cell Biol. 2015;17(9):1205-17. http://doi.org/10.1038/ncb3225

54. Tóth ML, Sigmond T, Borsos E, Barna J, Erdélyi P, Takács-Vellai K, et al. Longevity pathways converge on autophagy genes to regulate life span in Caenorhabditis elegans. Autophagy. 2008;4(3):330-8. http://doi.org/10.4161/auto.5618

55. Arriola AS, Pumper CP, Baar EL, Cummings NE, Lamming DW. Intermittent Administration of Rapamycin Extends the Life Span of Female C57BL/6J Mice. J Gerontol A Biol Sci Med Sci. 2016;71(7):876-81. http://doi.org/10.1093/gerona/glw064

56. Li SD, Ma M, Li H, Waluszko A, Sidorenko T, Schadt EE, et al. Cancer gene profiling in non-small cell lung cancers reveals activating mutations in JAK2 and JAK3 with therapeutic implications. Genome Med. 2017;9(1):89. http://doi.org/10.1186/s13073-017-0478-1

57. Xu Y, Jin J, Xu J, Shao YW, Fan Y. JAK2 variations and functions in lung adenocarcinoma. Tumour Biol. 2017;39(6):1393378524. http://doi.org/10.1177/1010428317711140

58. Xu M, Tchkonia T, Ding H, Ogrodnik M, Lubbers ER, Pirtskhalava T, et al. JAK inhibition alleviates the cellular senescence-associated secretory phenotype and frailty in old age. Proc Natl Acad Sci U S A. 2015;112(46):E6301-10. http://doi.org/10.1073/pnas.1515386112

59. Mayoux M, Roller A, Pulko V, Sammicheli S, Chen S, Sum E, et al. Dendritic cells dictate responses to PD-L1 blockade cancer immunotherapy. Sci Transl Med. 2020;12(534). http://doi.org/10.1126/scitranslmed.aav7431

60. Shikotra A, Ohri CM, Green RH, Waller DA, Bradding P. Mast cell phenotype, TNFα expression and degranulation status in non-small cell lung cancer. Sci Rep. 2016;6:38352. http://doi.org/10.1038/srep38352

61. Erfani N, Mehrabadi SM, Ghayumi MA, Haghshenas MR, Mojtahedi Z, Ghaderi A, et al. Increase of regulatory T cells in metastatic stage and CTLA-4 over expression in lymphocytes of patients with non-small cell lung cancer (NSCLC). Lung Cancer. 2012;77(2):306-11. http://doi.org/10.1016/j.lungcan.2012.04.011

62. Schneider T, Kimpfler S, Warth A, Schnabel PA, Dienemann H, Schadendorf D, et al. Foxp3(+) regulatory T cells and natural killer cells distinctly infiltrate primary tumors and draining lymph nodes in pulmonary adenocarcinoma. J Thorac Oncol. 2011;6(3):432-8. http://doi.org/10.1097/JTO.0b013e31820b80ca

63. Paz-Ares L, Ciuleanu TE, Cobo M, Schenker M, Zurawski B, Menezes J, et al. First-line nivolumab plus ipilimumab combined with two cycles of chemotherapy in patients with non-small-cell lung cancer (CheckMate 9LA): an international, randomized, open-label, phase 3 trial. Lancet Oncol. 2021;22(2):198-211. http://doi.org/10.1016/S1470-2045(20)30641-0

64. Bewersdorf JP, Shallis RM, Zeidan AM. Immune checkpoint inhibition in myeloid malignancies: Moving beyond the PD-1/PD-L1 and CTLA-4 pathways. Blood Rev. 2021;45:100709. http://doi.org/10.1016/j.blre.2020.100709

Supporting information

S1 Fig. A forest plot for 66 lncRNAs related to prognosis of LUAD.

S1 Table. 279 cellular senescence-related genes.

S2 Table. 62 cellular senescence-related DEGs.

S3 Table. 1081 cellular senescence-related lncRNAs.

S4 Table. Clinicopathological characteristics of LUAD patitents from TCGA.

S5 Table. 66 lncRNAs connected to the prognosis of LUAD.

S6 Table. 29 cellular senescence-related lncRNAs by LASSO.

S7 Table. 6 cellular senescence-related lncRNAs by multivariate Cox regression analysis.

S1 File. 6 lncRNAs recheck and calculations of coding potential

Fig1

Click here to access/download;Figure;Fig1.tif

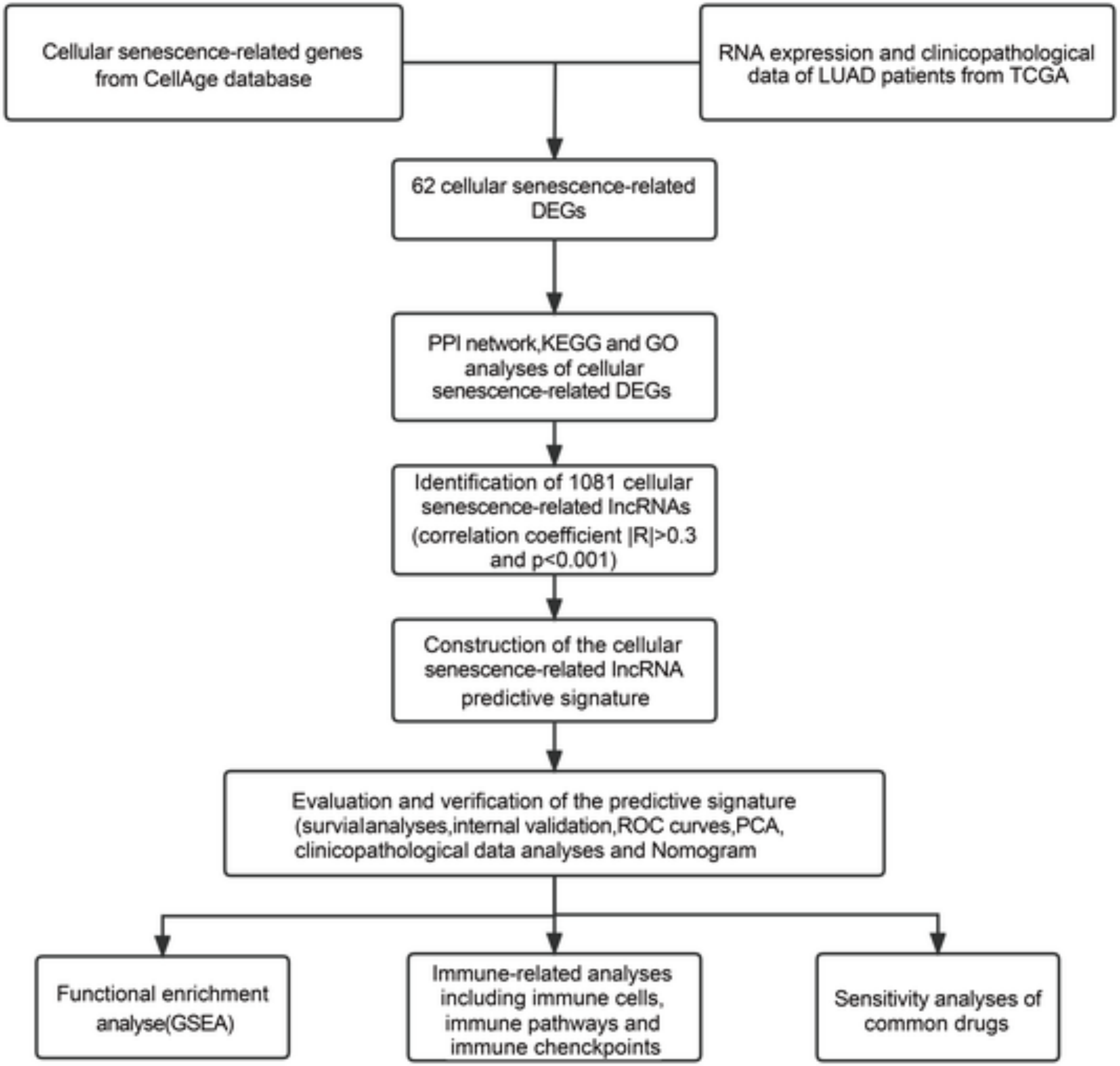

Fig2

Click here to access/download;Figure;Fig2.tif

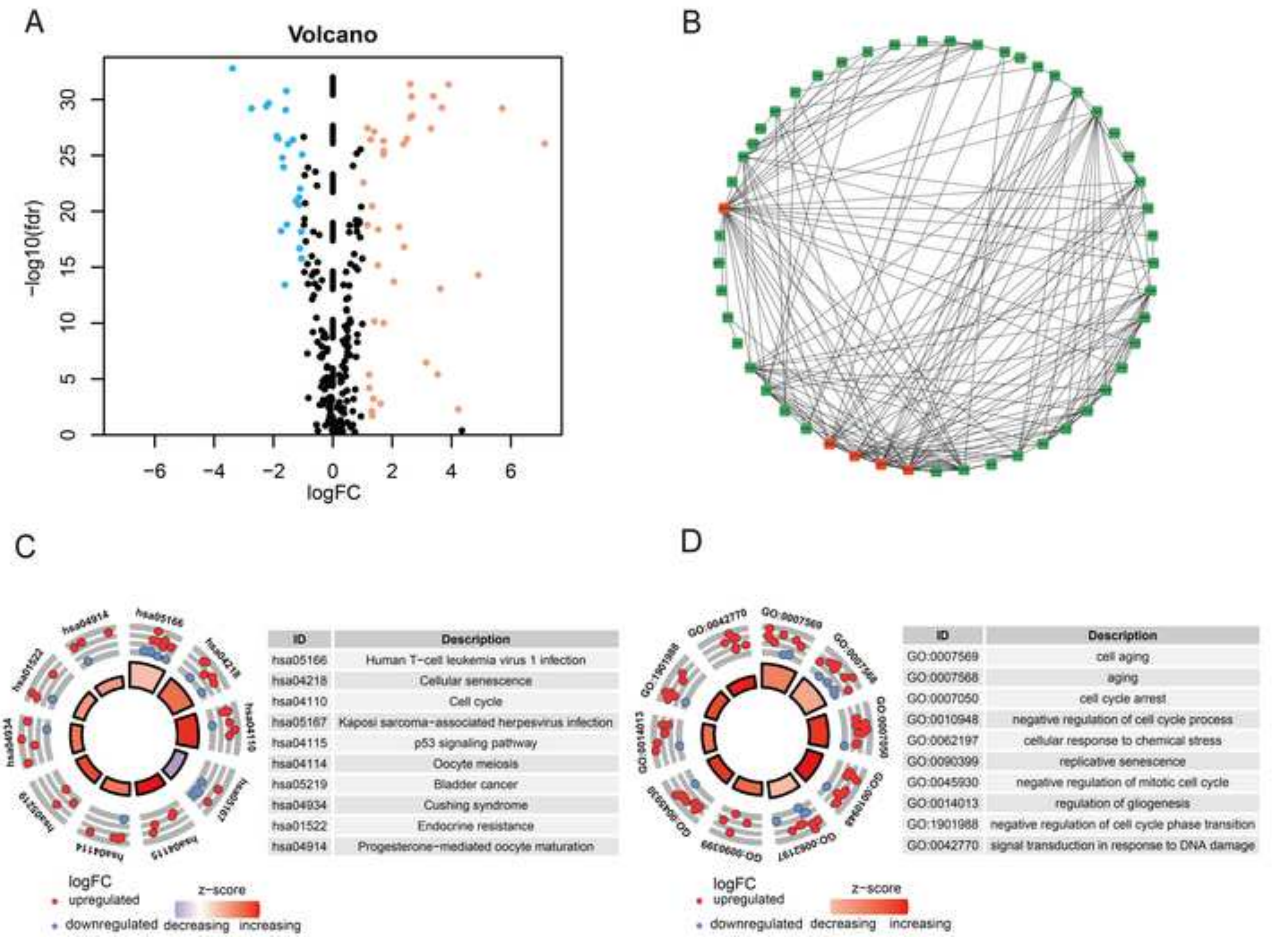

Fig3

Click here to access/download;Figure;Fig3.tif

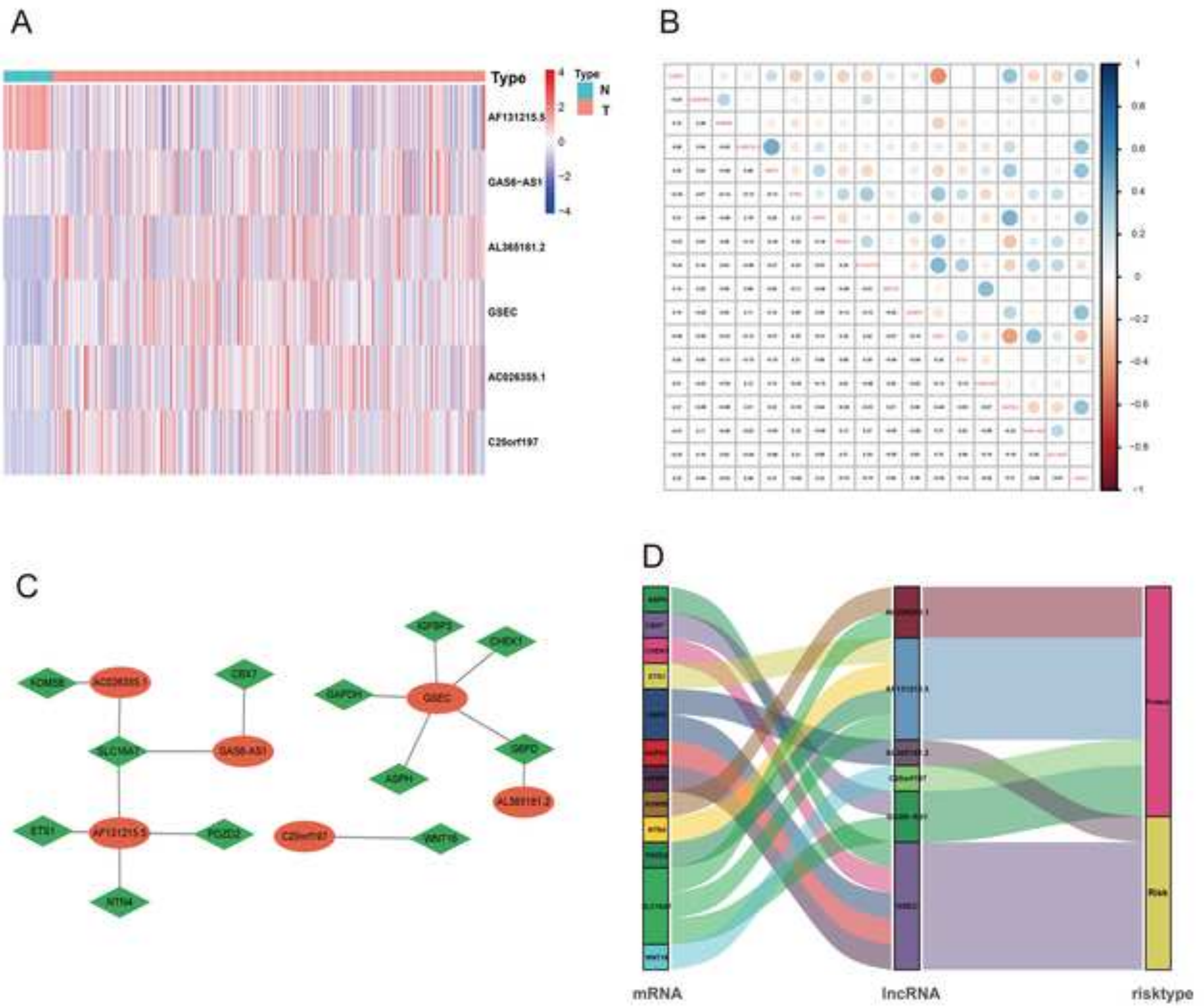

Fig4

Click here to access/download;Figure;Fig4.tif

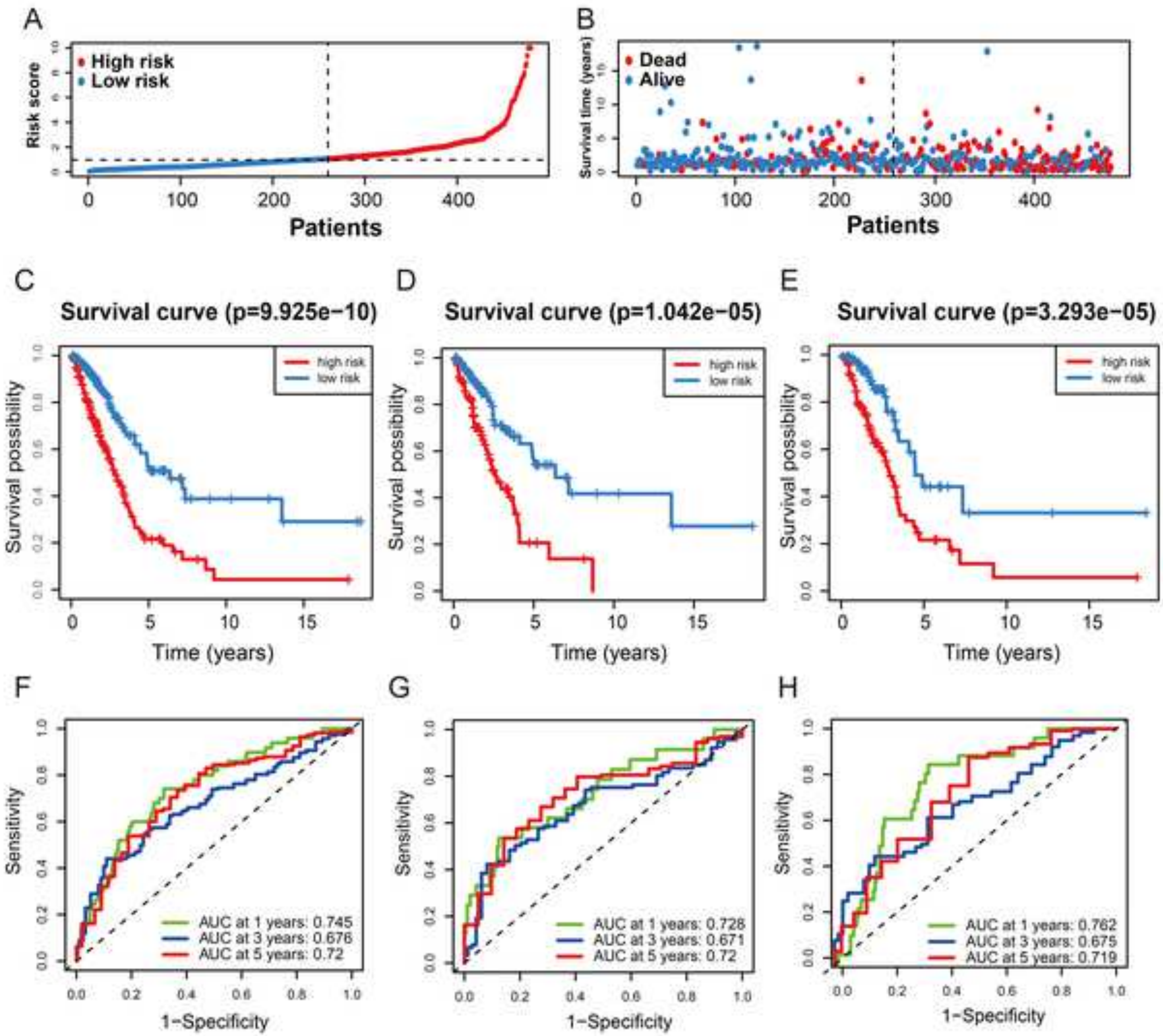

Click here to access/download;Figure;Fig5.tif

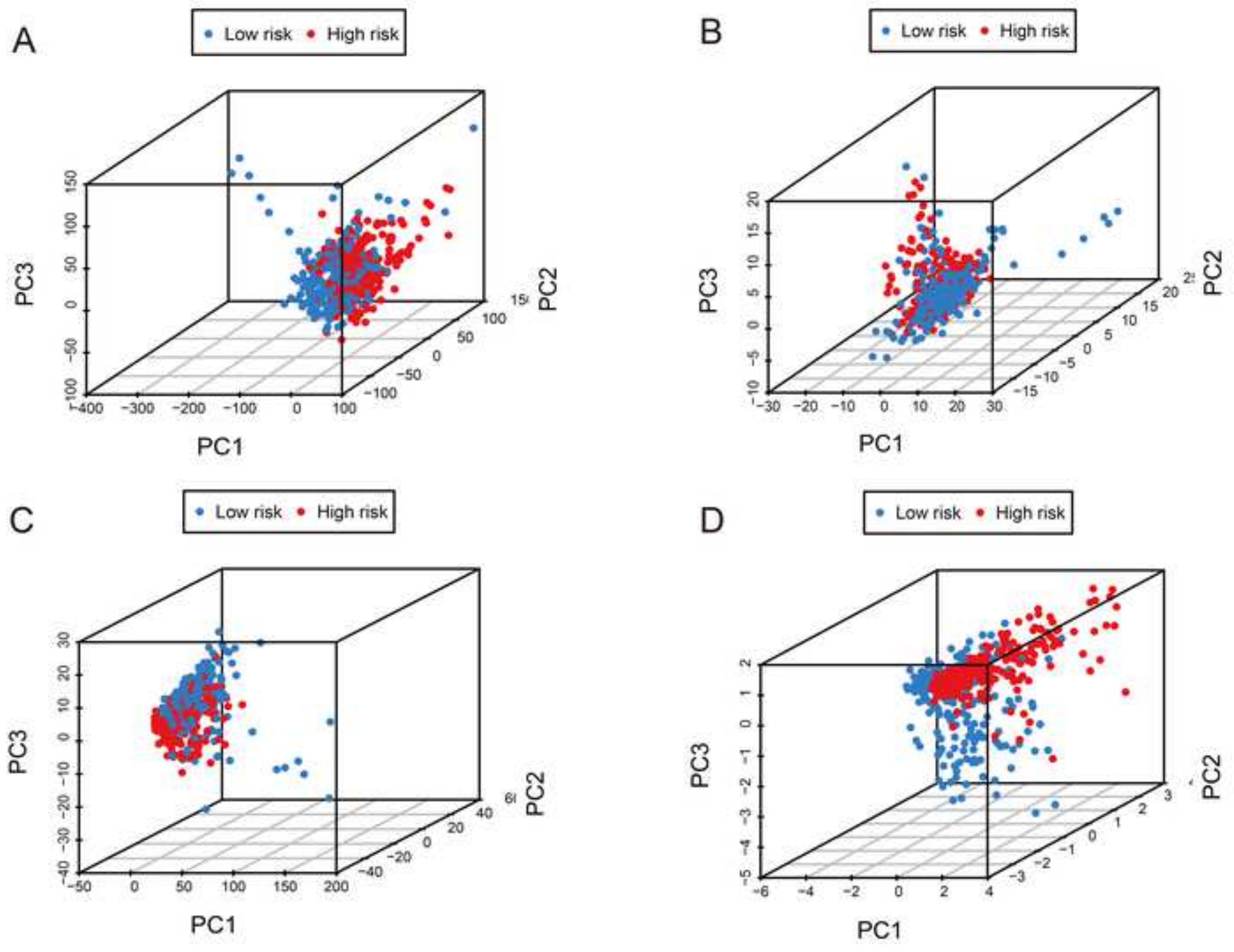

Fig6 Click here to access/download;Figure;Fig6.tif

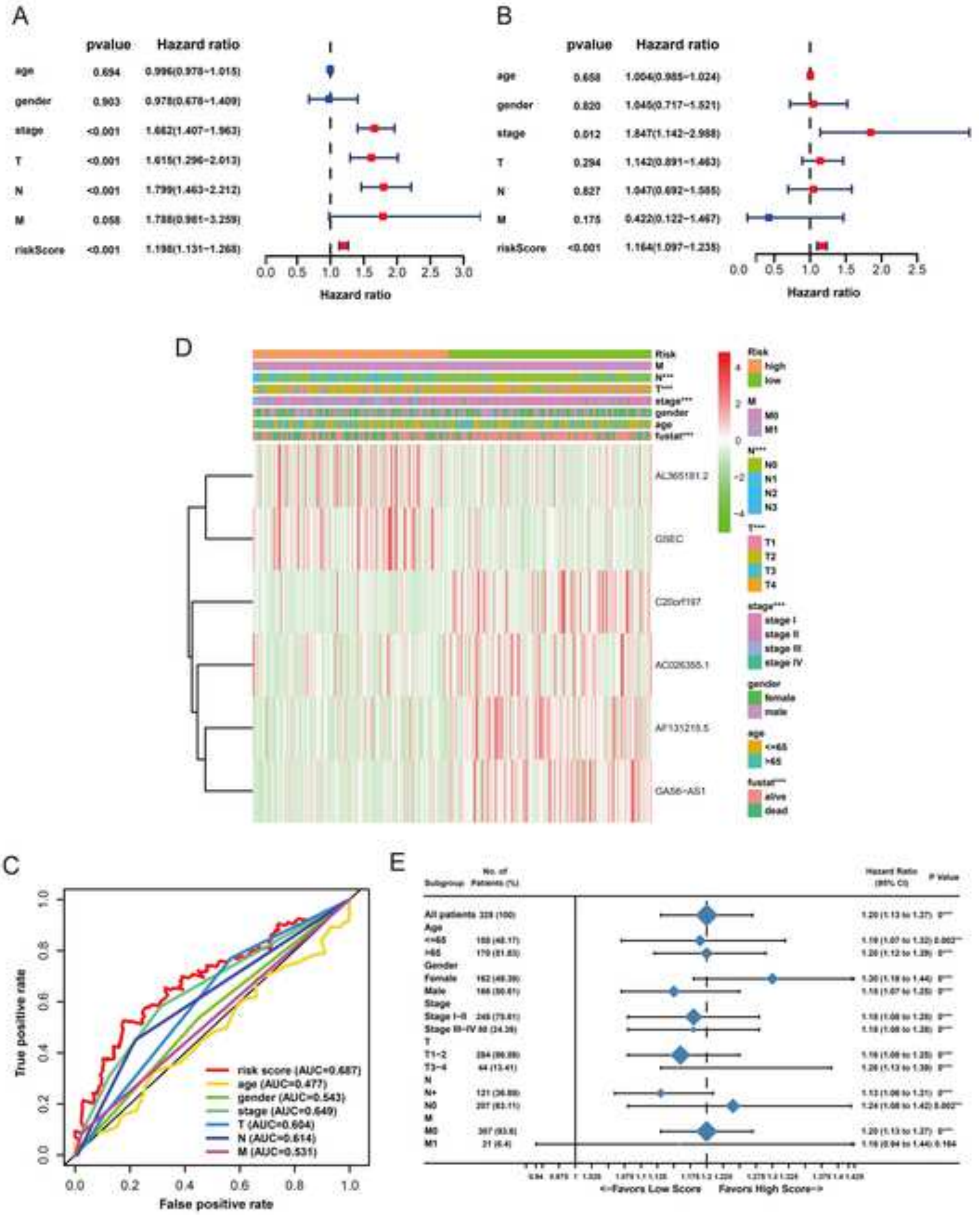

Fig7

Click here to access/download;Figure;Fig7.tif

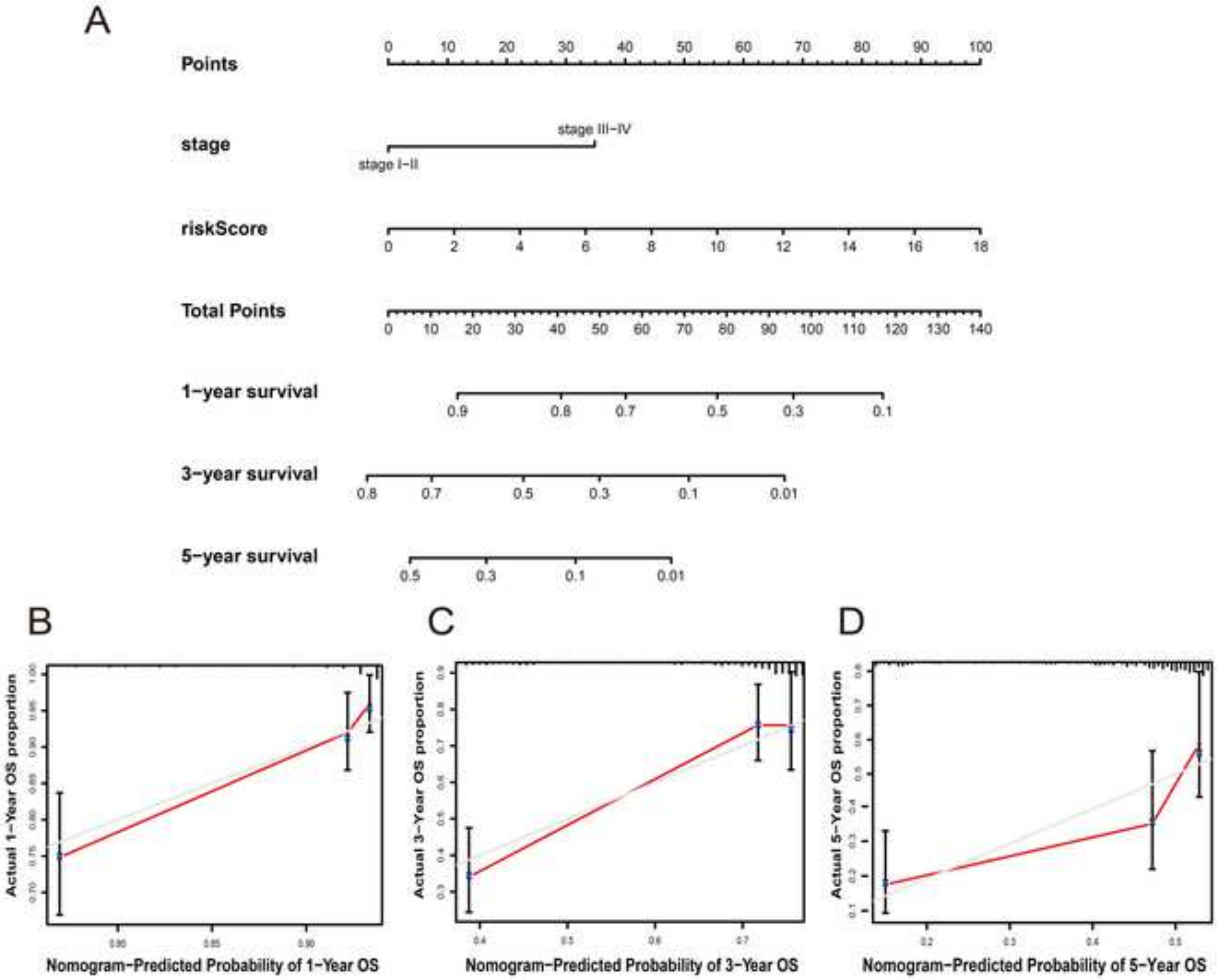

Click here to access/download;Figure;Fig8.tif

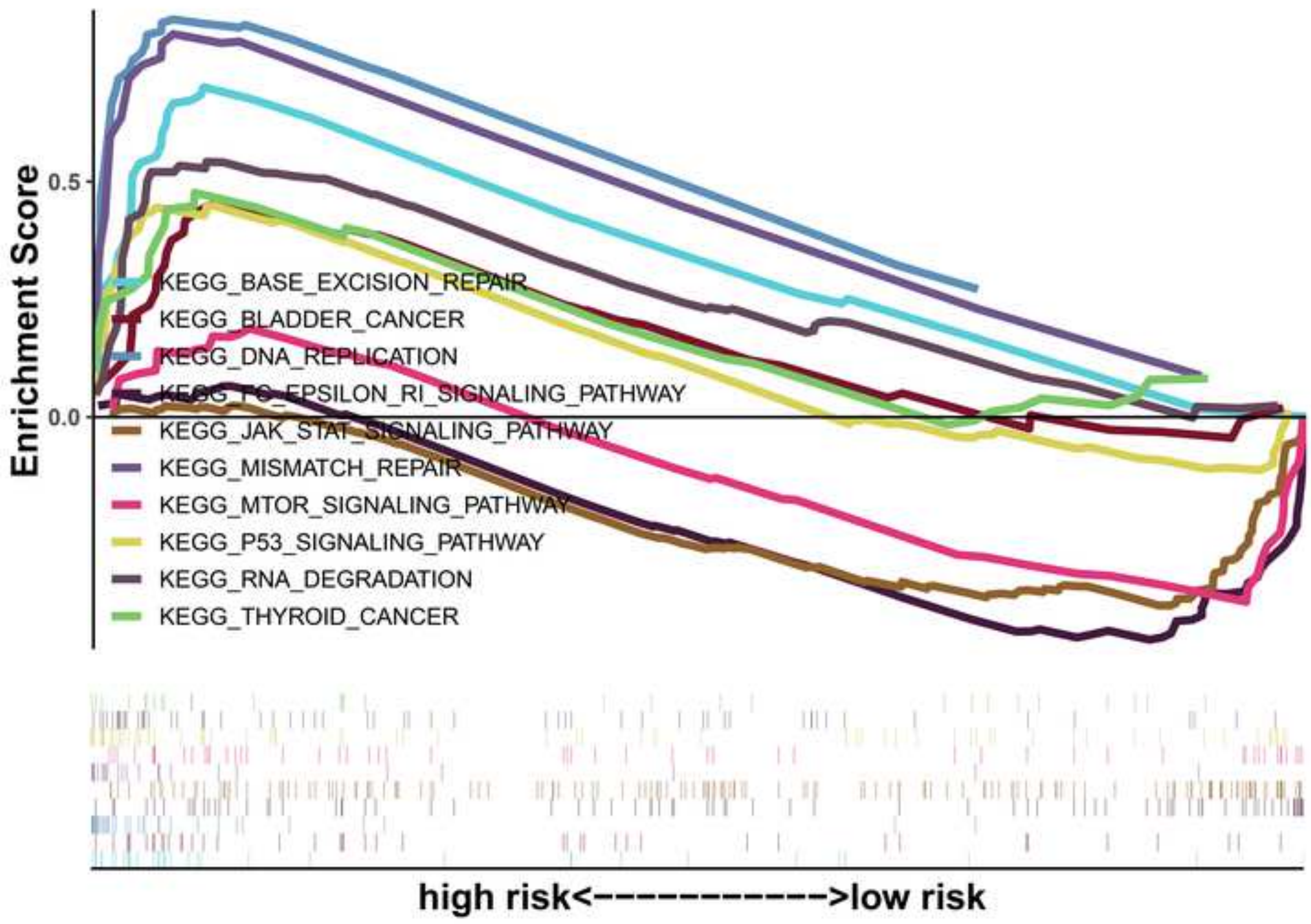

Fig9

Click here to access/download;Figure;Fig9.tif

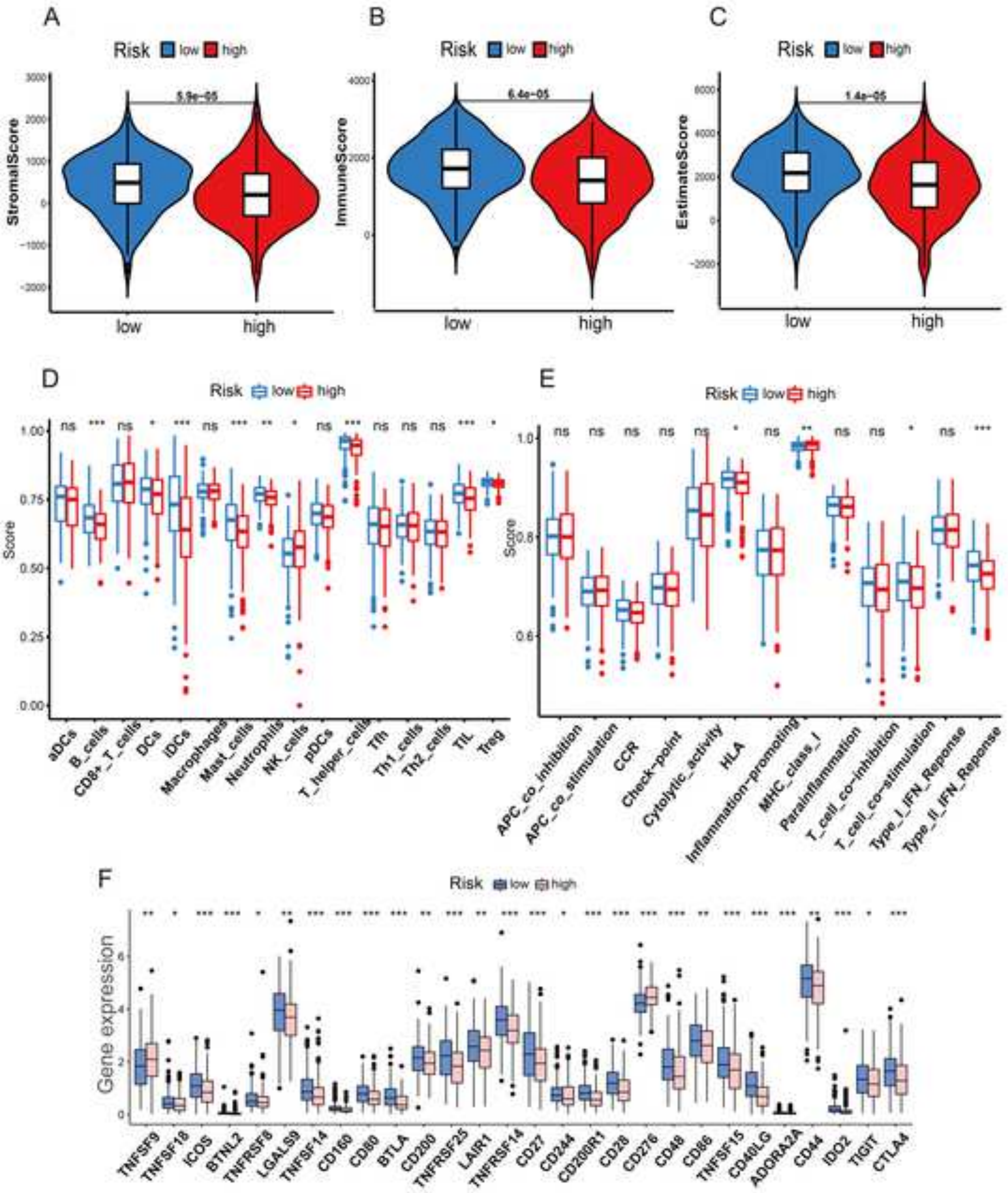

Click here to access/download;Figure;Fig10.tif

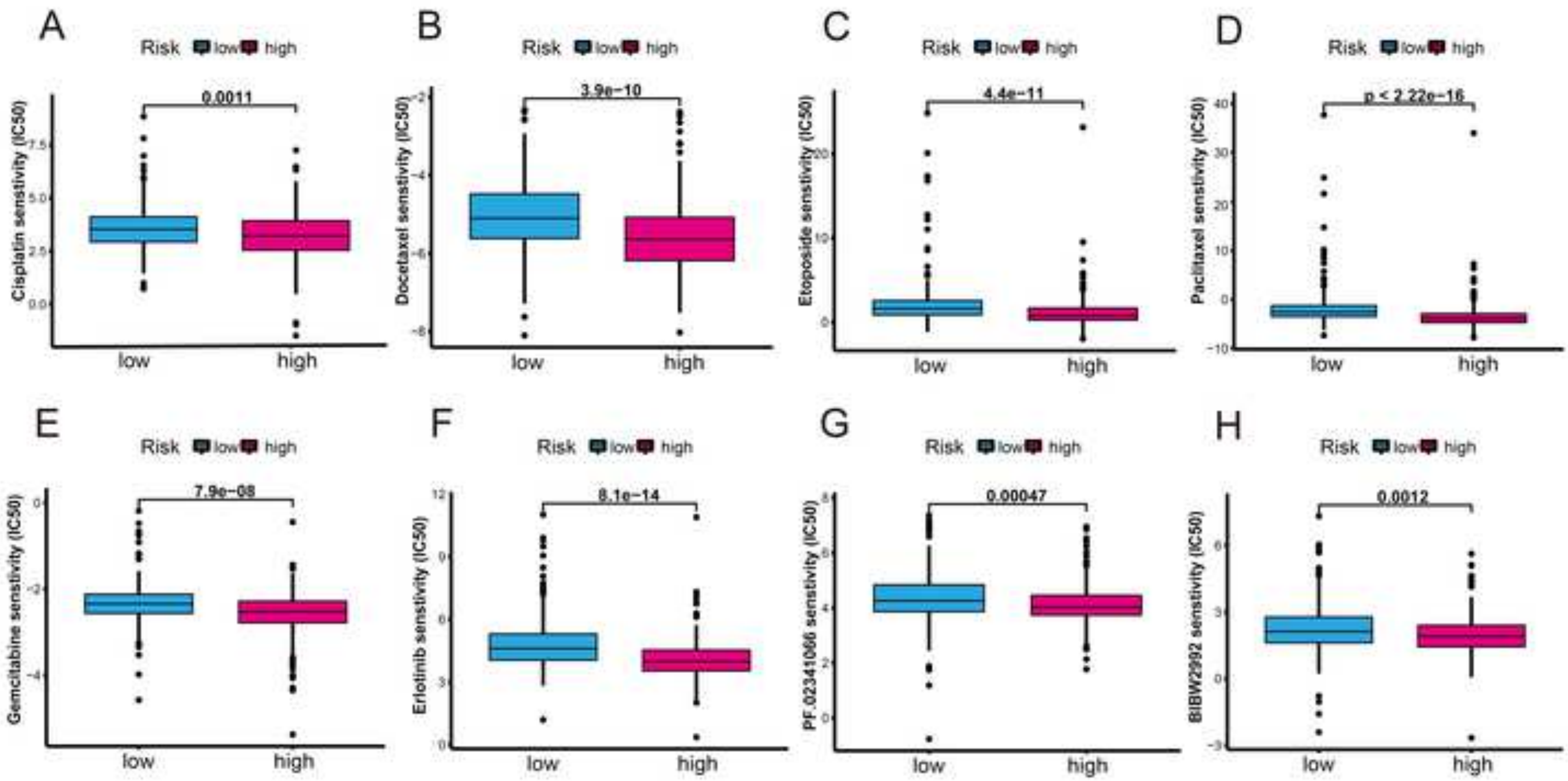

S1 Fig

Click here to access/download
Supporting Information
S1 Fig.pdf

S1 File

Click here to access/download
Supporting Information
S1 File.docx

S1 Table

Click here to access/download
Supporting Information
S1 Table.txt

S2 Table

Click here to access/download
Supporting Information
S2 Table.xls

S3 Table

Click here to access/download
Supporting Information
S3 Table.txt

S4 Table

Click here to access/download
Supporting Information
S4 Table.docx

S5 Table

Click here to access/download
Supporting Information
S5 Table.txt

S6 Table

Click here to access/download
Supporting Information
S6 Table.txt

S7 Table

Click here to access/download
Supporting Information
S7 Table.xls

~~A novel cellular senescence-related lncRNAs predictive signature for the prognosis of lung adenocarcinoma~~

A predictive molecular signature consisting of lncRNAs related to cellular senescence for the prognosis of lung adenocarcinoma

Anbang Liu[1¶], Xiaohuai Wang[1¶], Liu Hu[1], Dongqing Yan[1], Yin Yin[1], Hongjie Zheng[1], Gengqiu Liu[1], Junhang Zhang[1*] and Yun Li[1*]

[1]Department of Thoracic Surgery, The Seventh Affiliated Hospital, Sun Yat-sen University, Shenzhen, China

[*]liyun28@mail.sysu.edu.cn (YL)

[*]zhangjh33@mail.sysu.edu.cn (JZ)

[¶] These authors contributed equally to this work

Abstract

~~It has been established that various aging-related diseases can be influenced by cellular senescence, including cancer. There are few researches and reports on cellular senescence-related long noncoding RNAs (lncRNAs) in terms of lung adenocarcinoma (LUAD). To illustrate the prognostic value of cellular senescence-related lncRNAs, The Cancer Genome Atlas (TCGA) database was accessible to download the RNA sequencing (RNA-seq) dataset, survival~~

~~information, and associated clinicopathologic parameters of LUAD. The CellAge database provided the 279 genes involved in cellular senescence. We constructed a novel cellular senescence-related lncRNAs predictive signature (CS-RLPS). The CS-RLPS could be used to independently forecast the prognosis of LUAD patients and had a better predictive efficiency than other clinicopathological factors. Functional enrichment analysis of the CS-RLPS revealed that high-risk group mainly enriched in tumor-related pathways, whereas pathways associated with tumor-immune were enriched in low-risk individuals. According to immune-related analysis, the low-risk group had a closer connection with immune functions. Finally, sensitivity analyses of common drugs showed that high-risk group was more susceptible to the main chemotherapy and targeted therapy drugs. In conclusion, the CS-RLPS is a preferable prognostic indicator of LUAD patients. It is helpful to illustrate the potential molecular mechanism of cellular senescence-related lncRNAs, and provide appropriate strategies for the clinical treatment of patients with LUAD.~~

There are ~~few researches and~~ reports on cellular senescence-related long noncoding RNAs (lncRNAs) ~~in terms of~~ lung adenocarcinoma (LUAD). To explore the prognostic role of lncRNAs in LUAD, 279 cellular senescence-related genes, survival information and clinicopathologic parameters were ~~excavated~~ from the CellAge database and The Cancer Genome Atlas (TCGA) database. Then, we constructed a novel cellular senescence-related lncRNAs predictive signature (CS-RLPS) consisting of 6 lncRNAS (AC026355.1, AL365181.2, AF131215.5, C20orf197, GAS6-AS1, GSEC). According

to the median of the risk score, 480 samples were divided into high-risk and low-risk groups. Furthermore, the clinicopathological and biological functions, immune characteristics and common drug sensitivity were analyzed between two risk groups. In conclusion, the CS-RLPS can independently forecast the prognosis of LUAD, reveals the potential molecular mechanism of cellular senescence-related lncRNAs, and provides appropriate strategies for the clinical treatment of patients with LUAD.

Introduction

Globally, among all cancers, lung cancer ranks first in mortality and second in incidence nowadays[1]. Generally, there are two pathological types named non-small cell lung cancer (NSCLC) and small cell lung cancer. Lung adenocarcinoma (LUAD) is the most prevalent histological subtype of primary NSCLC, comprising roughly [illegible]0% [2]. According to the different stages of LUAD, patients need to receive comprehensive treatments including surgery, chemoradiotherapy, targeted therapy and immunotherapy, but the prognosis of advanced LUAD is still poor. Therefore, LUAD remains a serious public health problem that requires continuous attention, and it is indispensable to discover more drug targets, biomarkers and ~~improved~~ factors to lead to a better prognosis of patients.

Cellular senescence is characterized by a permanent cessation of cell proliferation. The occurrence of cellular senescence is associated with many different triggers, such as DNA damage and release of inflammatory cytokines, etc. Increasing evidence shows that many aging-related diseases can be prevented or delayed,

including cancer, by blocking-up the biological process of cellular senescence [3, 4]. A large of ~~researches~~ suggest that cellular senescence profoundly regulates tumor microenvironment by promoting the infiltration of some immune cells, activating many signaling pathways and releasing inflammatory cytokines [5-7]. Xue et al. found that in an in vivo model of hepatocellular carcinoma, restoration of *p53* could induce senescence of liver tumor cells and promote elimination of these cells by the innate immune system [8]. Some studies have found that *eIF3A R803K* mutation promotes resistance to chemotherapy by inducing tumor cell senescence and the higher expression of cellular senescence genes in human hepatocellular carcinoma is ~~closed~~ close to the ~~worse~~ worst overall survival (OS) [9, 10]. Furthermore, senescent thyroid cells can induce neighboring non-senescent thyroid cancer cells to invade and proliferate. The xenograft model also shows that senescent thyroid cells and cancer cells co-implanted increased lymphovascular invasion and lymph node metastasis through the CXCL12/CXCR4 axis [11].

Long noncoding RNA (LncRNA) refers to a kind of non-coding RNAs that are longer than 200nt and cannot encode proteins. LncRNA can regulate coding genes through chromatin remodeling, transcriptional control and post-transcriptional processing [12]. LncRNA mainly has four functions. The first function is "signaling function", which means the transcript of lncRNA itself serves as the marker of biological events including allelic specificity, structural specificity and various stimuli. Secondly, it is so-called "molecular decoy" that lncRNA can bind

with proteins and miRNAs, etc. The third is "guiding". LncRNA acts in cis or trans to target the bound effector molecule to the position of specific DNA sequence. Finally, lncRNA acts as a "molecular scaffold" can take part in the assembly of transcriptional regulatory complexes [13]. ~~Over the past decade,~~ On the basis of previous researches, it has been discovered that lncRNAs with aberrant expression contribute substantially to the onset and development of numerous diseases, ~~and~~ tumor growth and metastasis are particularly influenced by lncRNAs [14]. Several lncRNAs have the potential to be tumor biomarkers and therapeutic targets for the detection and treatment of cancer [15]. However, there are few researches and reports on cellular senescence-related lncRNAs in terms of LUAD.

Through this research, we constructed a cellular senescence-related lncRNAs predictive signature (CS-RLPS), and evaluated its prognostic value in LUAD patients. Furthermore, we further performed immune-related analyses and sensitivity analyses of common drugs to explore a more ~~reasonable~~ clinical treatment plan.

.Materials and Methods

~~LUAD dataset and cellular senescence-related gene set~~

Acquisition of LUAD dataset and cellular senescence-related gene set

The Cancer Genome Atlas (TCGA) database (https://portal.gdc.cancer.gov/) was accessible to download the RNA sequencing (RNA-seq) dataset, survival

information, and associated clinicopathologic parameters of LUAD. ~~The CellAge database (https://genomics.senescence.info/cells/) provided the 279 genes involved in cellular senescence (S1 Table).~~ Meanwhile, we obtained 279 cellular senescence-related genes from the CellAge database (https://genomics.senescence.info/cells/) (S1 Table).

Enrichment analyses of differentially expressed genes related to cellular senescence

We extracted the expression matrix of genes relevant to cellular senescence from the RNA-seq dataset, and used the “limma” package to draw the volcano plot of differentially expressed genes (DEGs). We considered the false discovery rate (FDR) < 0.05 and | log2 fold change (FC) | > 1 as the criteria of significantly differential expression, and further established a protein-protein interaction (PPI) network about DEGs through the STRING database (https://cn.string-db.org/). Then, we visualized the network by Cytoscape software, and screened out hub genes based on degree values by the application “cytoHubba”. The functions of DEGs were excavated by Gene Ontology (GO) and Kyoto Encyclopedia of Genes and Genomes (KEGG) analyses using the R package “clusterProfiler” and “GOplot” [16, 17]. ~~We used the R package “clusterProfiler” and “GOplot” to achieve the aim.~~

~~Construction and validation of the CS-RLPS~~

Construction, evaluation and verification of the predictive signature

We calculated the correlation between DEGs and lncRNAs by the “limma” package and regarded the correlation coefficient $|R| > 0.3$ and $p < 0.001$ as screening standard. ~~Furthermore, we identified 1081 cellular senescence-related lncRNAs.~~ In order to investigate the connection between cellular senescence-related lncRNAs and the OS, the univariate Cox regression analysis was initially performed. Least absolute shrinkage and selection operator (LASSO) was further used to obtain the cellular senescence-related lncRNAs through the “glmnet” package and taking 10-fold cross-validation [18, 19]. Lastly, we used the multivariate Cox regression analysis to identify and construct an efficient CS-RLPS on the basis of the following computational formula:

$$\text{Risk score} = \sum_{i=1}^{n} \text{Coef(i)} \times \text{Expr(i)}$$

~~Coef(i) and Expr(i) respectively represent the multivariate Cox regression coefficient for each cellular senescence-related lncRNA and corresponding lncRNA expression level.~~

Coef(i) and Expr(i) represent the multivariate Cox regression coefficient and corresponding lncRNA expression level, respectively. We could calculate the risk score for each LUAD patient using the formula. Furthermore, on the basis of the median risk score, we divided LUAD patients into two categories, named respectively high-risk and low-risk groups.

We tested the reliability of the CS-RLPS by principal component analysis (PCA). Additionally, through the “rms” package, we made a nomogram for the purpose of

predicting LUAD survival at 1-year, 3-year, and 5-year. The performance of the nomogram was evaluated using calibration curve.~~ The calibration curves were further to verify whether the CS-RLPS had good forecasting efficacy predictive performance.~~

~~Enrichment Analysis of the CS-RLPS~~

Gene set enrichment analysis (GSEA) 4.1.0 could help to investigate which pathways were mostly enriched in high-risk and low-risk groups, respectively. [20]. Normalized enrichment score |NES| ≥ 1 and nominal $p < 0.05$ were taken into account as our standard for statistical significance.

Immune-related analyses of the CS-RLPS

~~We performed immune-related analyses of the CS-RLPS,~~ Immune-related analysis was performed for the CS-RLPS, including the infiltration of stromal and immune cell. We calculated the StromalScore, ImmuneScore, and ESTIMATEScore respectively by the R package "estimate" and the Wilcoxon signed-rank test was applied to compare immune scores between the different risk groups[21]. Following that, ~~through single-sample gene set enrichment analysis (ssGSEA), the infiltration scores of 16 immune cells were computed and the biological functions of 13 immune-related pathways were explored~~. Single-sample gene set enrichment analysis (ssGSEA) was used to compute the infiltration scores of immune cells and explore the biological functions of immune-related pathways between high- and low-risk groups through the "GSVA" and "GSEABase" packages[22].

~~Predicting Efficacy of LUAD Therapy~~

Sensitivity analysis of common drugs

To explore whether the CS-RLPS was a guidance for LUAD therapy, we chose the half-maximal inhibitory concentration (IC50) as a reference to evaluate the predictive role of the signature in chemotherapy and targeted therapy. IC50 of some common drugs for chemotherapy and targeted therapy of LUAD were calculated by the R package “pRRophetic” [23].

we obtained the half-maximal inhibitory concentration (IC50) of some conventional chemotherapy drugs and targeted drugs as pharmacological data. We chose the half-maximal inhibitory concentration (IC50) as a reference. The value of IC50 could reflect the sensitivity to chemotherapy and targeted therapy in two different risk groups.to evaluate the predictive role of the CS-RLPS in chemotherapy and targeted therapy. IC50 of some common drugs for chemotherapy and targeted therapy of LUAD were calculated by the R package “pRRophetic” [23]. Here, the Wilcoxon signed-rank test was utilized to compare the IC50 values of different risk groups.

Statistical analysis

R software (Version 4.0.5) was carried out conducting all statistical analyses. If not otherwise stated, $p < 0.05$ was thought to be statistically meaningful. The expression levels of DEGs related to cellular senescence were shown by Wilcoxon signed-rank test. The R package "survival" performed the OS analyses of patients with LUAD through the Kaplan-Meier method. The Wilcoxon signed-rank test was utilized to compare differences between two groups. The Kaplan–Meier method was employed for survival analyses by the R package “survminer”and “survival”. We used the “timeROC” and “survivalROC” package to generate the time-dependent and

conventional ROC curves. According to the area under the curves (AUCs), the predictive efficacy could be determined.

Results

~~Enrichment Analyses of the DEGs~~

Identification and analyses of the cellular senescence-related DEGs

Fig 1 depicts a flow diagram of our work. We obtained 62 cellular senescence-related DEGs, comprising 39 upregulated and 23 downregulated genes (Fig 2A, S2 Table). ~~On the basis of 62 DEGs associated with cellular senescence, the STRING online database was utilized to build a PPI network that was further displayed by Cytoscape software~~. According to the visual PPI network, we discovered the five most valuable genes as the hub genes based on their degree values (Fig 2B). As was shown in KEGG analyses, the enrichment of DEGs was most prominent along these pathways, such as cellular senescence, cell cycle, the p53 signaling pathway, oocyte meiosis, bladder cancer, cushing syndrome, endocrine resistance and progesterone−mediated oocyte maturation (Fig 2C). Then, GO analyses revealed that these DEGs involved in the cell aging, cellular response to chemical stress, the replicative senescence, negative regulation of mitotic cell cycle, regulation of gliogenesis and so on (Fig 2D).

Fig 1. The flow diagram of our research.

Fig 2. Enrichment analyses. (**A**) The volcano plot of 62 cellular senescence-related DEGs. (**B**) The PPI network of cellular senescence-related DEGs, red squares

represent top 5 hub genes. (**C**, **D**) KEGG and GO analysis of cellular senescence-related DEGs, respectively.

~~Construction of the CS-RLPS~~

Construction and internal validation of the CS-RLPS

We screened out 1081 cellular senescence-related lncRNAs (S3 Table). Moreover, the 480 LUAD patients including both RNA expression data and survival data were acquired from TCGA dataset ~~which both whose expression data and survival data were available~~, and the OS of these samples was more than 30 days old (S4 Table). 66 lncRNAs were connected to the prognosis of LUAD following the outcomes of the univariate Cox regression analysis **(**S1 Fig, S5 Table**)**. Then, LASSO ~~found out~~ identified that 29 lncRNAs were involved in cellular senescence and the multivariate Cox regression analysis identified 6 cellular senescence-related lncRNAs (*AC026355.1*, *AL365181.2*, *AF131215.5*, *C20orf197*, *GAS6-AS1*, *GSEC*) **(**S6 and S7 Tables, S1 File**)**. Finally, we ~~and~~ constructed the CS-RLPS according the above analysis. The expression ~~value~~ levels of 6 cellular senescence-related lncRNAs ~~was~~ were displayed as a heatmap ~~in LUAD patients~~ (Fig 3A). Fig 3B showed the correlation ~~analysis~~ between 6 cellular senescence-related lncRNAs and 12 co-expressed mRNAs. The co-expression network of 15 pairs of lncRNA-mRNA was ~~showed~~ shown in Fig 3C ($| R | > 0.3$ and $p < 0.001$). Among these pairs, *GSEC* was found to be co-expressed with 5 cellular senescence-related genes (*IGFBP3*, *CHEK1*, *G6PD*, *GAPDH* and *ASPH*)~~,~~. *AF131215.5* had co-

expressed relationship with 4 cellular senescence-related genes (*ETS1*, *NTN4*, *PDZD2* and *SLC16A7*)~~;~~. *GAS6-AS1* had co-expressed relationship with *CBX7* and *SLC16A7*~~;~~. *AL513550.1* had co-expressed relationship with *KDM5B* and *SLC16A7*~~;~~. *AL365181.2* was co-expressed with *G6PD*~~;~~. *C20orf197* was co-expressed with *WNT16*. We also found that *AL365181.2* and *GSEC* were risk factors, however, the other four lncRNAs were protective factors (*AC026355.1*, *AF131215.5*, *C20orf197*, *GAS6-AS1*) (Fig 3D). We designed a formula used for risk score. Risk score = (-0.270×*AC026355.1* expression, 0.231×*AL365181.2* expression, -0.616×*AF131215.5* expression, -0.580×*C20orf197* expression, -0.627×*GAS6-AS1* expression, 0.505×*GSEC* expression).

Fig3. Construction of the CS-RLPS. (**A**) The heatmap of 6 cellular senescence-related lncRNAs in tumor and normal tissues. (**B**) The correlation analysis between 6 cellular senescence-related lncRNAs and 12 co-expressed mRNAs. (**C**) The co-expression network of lncRNAs and mRNAs. (**D**) The Sankey diagram for cellular senescence-related lncRNAs, mRNAs and risk status. T, tumor; N, normal.

Correlation between the CS-RLPS and LUAD prognosis

We obtained the median after determining the risk score for each sample using the formula. Subsequently, all samples were divided into high- and low-risk groups based on the median of risk score. ~~we divided the samples into made a distinction between the high- and low-risk groups through based on the median~~. The risk scores of two groups were shown in Fig 4A. As the scores climbed, so did the likelihood

of death (Fig 4B). Compared with those at high risk, the OS of low-risk patients was noticeably longer (Fig 4C, p=9.925e−10). The AUCs of 1-year, 3-year, and 5-year survival were respectively 0.745, 0.676 and 0.72, which manifested that the signature had relatively good predictive performance (Fig 4F). In order to verify the applicability of CS-RLPS, afterwards, two internal validation cohorts were formed by randomly dividing the LUAD patients. The results of the OS were consistent with the total cohort of predictive signature (Fig 4D, p=1.042e−05; Fig 4E, p=3.293e−05). Meanwhile, the two validation cohorts both showed ~~a~~ similar predictive performance to the total cohort according to the ROC curves. The AUCs of 1-year, 3-year and 5-year survival were respectively 0.728, 0.671 and 0.72 in the first cohort (Fig 4G) and those of the second cohort were respectively 0.762, 0.675 and 0.719 (Fig 4H). PCA 3D plots were used to distribute LUAD patients based on the entire genome, gene sets associated with cellular senescence, cellular senescence-related lncRNAs, and the CS-RLPS (Figs 5A-D). The result identified that the CS-RLPS obviously discriminated ~~degree of~~ risk degree in different quadrants. On the contrary, the other groups could not be effectively distinguished, further supporting the reliability of the CS-RLPS.

Fig4. Correlation between the CS-RLPS and LUAD prognosis. (**A**) The distribution of LUAD patients. (**B**) A ~~scatterplot~~ scatter plot based on each sample's OS and risk score. (**C-E**) The survival curves of LUAD patients in the total cohort and two internal cohorts, respectively. (**F-H**) The ROC curves for the total cohort and the two internal cohorts at 1-year, 3-year, and 5-year, respectively.

~~Principal Component Analysis~~

~~PCA 3D plots were used to distribute LUAD patients based on the entire genome, gene sets associated with cellular senescence, cellular senescence-related lncRNAs, and the CS-RLPS (Figs 5A-D). The result identified that the CS-RLPS obviously discriminated degree of risk in different quadrants. On the contrary, the other groups could not be effectively distinguished, further supporting the reliability of the CS-RLPS.~~

Fig5. PCA 3D plots based on (**A**) the entire genome, (**B**) the gene sets associated with cellular senescence, (**C**) the cellular senescence-related lncRNAs, (**D**) the CS-RLPS.

Relationship between the CS-RLPS and clinicopathological factors

The univariate Cox regression analysis revealed that stage, T, N and the CS-RLPS were closely connected to the OS of LUAD (Fig 6A). Stage and the CS-RLPS were identified as two independent predictors after multivariate Cox regression analysis ~~determined~~ (Fig 6B). Fig 6C indicated that the best predictor among all factors was the risk score in LUAD because of the largest AUC（AUC=0.687）. The differences of all clinicopathological factors were further studied, and the result revealed ~~found~~ that N, T, stage and survival state were statistically different from the other factors between the two risk groups (Fig 6D). Next, we obtained the data of 328 LUAD patients including all expression data, survival data and clinicopathological information to investigate the relationship between the CS-RLPS and patients' prognosis. These samples were divided into different clinical subgroups on

the basis of different clinicopathological factors. The results indicated that CS-RLPS as a risk factor was a relatively better predictor for LUAD in different clinicopathological subgroups except M1 subgroup ~~group~~ (Fig 6E).

Fig6. Relationship between the CS-RLPS and clinicopathological factors. Forest plots for (**A**, **B**) The univariate and multivariate Cox regression analyses of the CS-RLPS and clinicopathological factors. (**C**) The ROC curve of the CS-RLPS and clinicopathological factors. (**D**) Distribution heatmap of 6 lncRNAs and clinicopathological factors. (**E**) A forest plot for clinicopathological subgroup analysis. T, tumor; N, lymph node; M, metastasis. *p < 0.05; **p < 0.01; ***p < 0.001.

~~Nomogram and Calibration~~

The nomogram predicted the prognosis of LUAD

We further constructed a nomogram including stage and the CS-RLPS, which was able to forecast LUAD survival for 1-year, 3-year, and 5-year (Fig 7A). For the sake of evaluating the predictive efficacy of the signature, the calibration curves were performed and~~. The findings~~ showed that the predictive survival rates were fairly consistent with ~~and~~ the actual survival rates at 1-year, 3-year, and 5-year ~~were fairly consistent~~ (Figs 7B–D).

Fig7. Nomogram and Calibration. (**A**) A nomogram of predicting LUAD survival at 1-year, 3-year, and 5-year. (**B-D**) The calibration curves for predictive accuracy of the CS-RLPS.

Functional enrichment analysis of the CS-RLPS

Enrichment analysis of the CS-RLPS was further carried out by GSEA to probe the underlying functions between two risk groups (Fig 8). The results indicated that the high-risk patients were connected to tumor-associated pathways, such as DNA replication (NES =2.03 and nominal p <0.001), RNA degradation (NES = 1.81 and nominal p = 0.008), p53 signaling pathway (NES = 1.69 and nominal p = 0.013) and so on. The results also revealed that the low-risk group was closely associated with tumor-immune related pathways, such as FC epsilon RI signaling pathway (NES = -1.86 and nominal p = 0.004), mTOR signaling pathway (NES = -1.63 and nominal p =0.006) and JAK/STAT signaling pathway (NES = -1.62 and nominal p = 0.031). The above results fully proved that cellular senescence-related lncRNAs were linked to tumor immune microenvironment (TIME) in our predictive signature.

Fig8. The signaling pathways of significant enrichment according to GSEA.

~~Immune-related Analyses~~

Relationship between the CS-RLPS and immune characteristics

The three violin plots respectively showed StromalScore, ImmuneScore and ESTIMATEScore in two risk groups. Notably, the three types of scores were all significantly higher in low-risk patients (Figs 9A–C). To further investigate the

association between the CS-RLPS and immune functions, we assessed the ssGSEA enrichment scores for several immune cell subgroups and immune-related pathways. The analysis showed that most of the immune cell infiltration happened in the low-risk group, including T regulatory cells (Tregs), tumor-infiltrating lymphocyte (TIL), T helper cells, neutrophils, mast cells and immature dendritic cells (iDCs) (Fig 9D). There were four immune-related pathways significantly associated with the CS-RLPS, including type II Interferon (IFN) response, T cell co-inhibition, major histocompatibility complex (MHC) class I, and human leukocyte antigen (HLA) (Fig 9E). Furthermore, we discovered that the genes related to immune checkpoint were highly expressed in the low-risk group~~ patients at low risk~~, particularly, such as *ICOS*, *BTNL2*, *TNFSF14*, *CD160*, *CD80*, *BTLA*, *TNFRSF25*, *TNFRSF14*, *CD27*, *CD200R1*, *CD28*, *CD48*, *TNFSF15*, *CD40LG*, *ADORA2A*, *IDO2* and *CTLA4*, etc. (Fig 9F).

Fig9. Immune-related Analyses. (**A-C**) The violin plots of StromalScore, ImmuneScore, and ESTIMATEScore between two groups with high-and low-risk. (**D**, **E**) The enrichment scores of immune cells and immune-related pathways between two groups with high-and low-risk. (**F**) The expression of immune checkpoint between two groups with high-and low-risk. T, tumor; N, normal; M, metastasis; ns, non-significant; *p < 0.05; **p < 0.01; ***p < 0.001.

~~Correlation Between the CS-RLPS and LUAD Therapy~~

Predicting efficacy of the CS-RLPS in LUAD therapy

To explore whether the CS-RLPS was a guidance for LUAD therapy, we obtained the IC50 of some conventional chemotherapy drugs and targeted drugs as pharmacological data. The results showed that the IC50 of cisplatin, docetaxel, etoposide, paclitaxel, gemcitabine, erlotinib, crizotinib and afatinib was lower in ~~those at high risk~~ high-risk group, meaning that the high-risk individuals were more sensitive to the majority of common chemotherapy drugs and targeted drugs (Figs 10A-H). Therefore, the CS-RLPS could be used for predicting the efficacy of LUAD therapy and provided appropriate clinical guidance.

Fig10. The sensitivity analysis of common chemotherapeutic and targeted drugs. (**A-E**) The chemotherapy drugs including Cisplatin, Docetaxel, Etoposide, Paclitaxel, Gemcitabine. (**F-H**) The targeted drugs including Erlotinib, PF.02341066 (Crizotinib) and BIBW2992 (Afatinib).

Discussion

Currently, LUAD is the most epidemic pathological type that threatens non-smokers, and its incidence is persistently increasing year by year. Even though there are a host of treatments available, the prognosis of advanced LUAD remains very poor [24]. With the deepening research on tumor suppressor mechanism, the prevention of tumorigenesis and development by inducing tumor cellular senescence is gradually receiving attention. Cellular senescence is an anti-proliferative program that leads to permanent cell growth arrest [25] and protects cells from unnecessary damage [26].

In this study, we constructed a CS-RLPS for predicting the prognosis of LUAD. We obtained 62 cellular senescence-related DEGs, of which 5 genes were most closely associated with the other genes. Among them, *CDKN2A* encodes a nucleolar protein involved in triggering cell cycle arrest and apoptosis to suppress tumor growth [27]. Studies have shown that *CDKN2A* represses *MDM2*, a negative regulator of *p53*, thereby stabilizing *p53* and enabling it to generate a transcriptional response by relocating it to the nucleolus [28]. Liu et al. found that *CDKN2A* loss promoted the progression of lung cancer and was closely associated with poorer survival outcomes [29]. Cyclin dependent kinase 1 (*CDK1*) is one of the most important factors regulating the cell cycle, which is crucial for the time phase transitions of the cell cycle. Multiple bioinformatics analyses suggest that *CDK1* expressed highly in LUAD, negatively correlated to the OS of individuals with LUAD [30-32]. Li et al. showed that *miR-143-3p* could target and regulate *CDK1* and promote cancer cell apoptosis in LUAD A549 cell line [33].

In the enrichment analyses of 62 DEGs, we were pleasantly surprised to find that there was a significant enrichment in the *p53* pathway. A great deal of genes mediating cell cycle arrest can be regulated via *p53* signaling pathway [34]. The pathway is also involved in regulating cellular senescence, which is consistent with our enrichment results [34, 35]. Previous evidence has shown that the *p53* pathway can accelerate apoptosis and autophagy of LUAD cells [36, 37]. ~~Based on enrichment results, we believe~~ that cellular senescence-related genes can inhibit the biological behavior of LUAD through the *p53* pathway.

The role of lncRNAs in LUAD has been verified by more and more ~~researches.~~ However, there are few reports on the role of lncRNAs related to cellular senescence in LUAD patients. Roth et al. indicated that down-regulating the expression of *linc00673* could trigger the senescence of LUAD cells, thereby exerting an inhibitory effect on LUAD [38]. Tao et al. confirmed that lncRNA *MEG3* promoted etoposide-induced tumor cell senescence through the *miR-16-5p*/*VGLL4* axis [39]. In our study, we ultimately identified 6 cellular senescence-related lncRNAs (*AF131215.5*, *GAS6-AS1*, *AC026355.1*, *GSEC*, *AL365181.2*, *C20orf197*) through multivariate COX regression analysis for building the predictive signature. We also found that 12 mRNAs (*ETS1*, *CBX7*, *SLC16A7*, *NTN4*, *PDZD2*, *KDM5B*, *WNT16*, *ASPH*, *IGFBP3*, *CHEK1*, *G6PD*, *GAPDH*) co-expressed with these 6 lncRNAs. Among them, high expression of NTN4 attenuates DNA damage-induced senescence in Glioblastoma multiforme (GBM) [40]. *PDZD2* is able to induce senescence in prostate cancer, breast cancer and liver cancer cells via the *p53* signaling pathway [41]. Knockdown of *GAPDH* can trigger cellular senescence in A549 cell line. Then, further experiments have found that *GAPDH* depletion may induce and accelerate cellular senescence of tumor through the *AMPK* network in the absence of DNA damage [42]. Besides, high expression of *G6PD* can also promote the growth of glioblastoma and inhibit its senescence [43].

In this research, LUAD patients were categorized as ~~either~~ high ~~or~~ and low risk according to the median risk score. The ROC curve suggested that the CS-RLPS had relatively outstanding predictive efficacy. Moreover, we randomly divided these

patients into two internal validation cohorts and plotted ROC curves separately. The results suggested that they had the semblable predictive performance as the total cohort. The analysis of clinicopathological factors led to the conclusion that the CS-RLPS could be regarded as an independently prognostic indicator of LUAD patients. GSEA showed that JAK/STAT pathway, mTOR pathway and FC epsilon RI pathway was enriched notably in the low-risk group. Thereinto, FC epsilon RI signaling pathway has many connections with immune function [44], while mTOR signaling pathway and JAK/STAT signaling pathway are closely associated with cancers. Many researches have indicated that the mTOR signaling pathway is one of the most common regulatory pathways which affected the occurrence, development and metastasis of LUAD by inducing cell apoptosis [45-47]. Rapamycin and temsirolimus as the mTOR inhibitors have already been used to treat NSCLC in various phases of clinical trials [48, 49]. Currently, in accordance with more and more genetic and pharmacological evidence, mTOR activity is recognized as a main driver of cellular senescence [50]. Carroll B et al. found that *mTORC1* hyperactivity was an obvious characteristic of cellular senescence, which could arrest the cell cycle [51]. Moreover, it was found that senescent cells produced pro-inflammatory cytokines to exacerbate aging-related tissue function decline by utilizing translational programs downstream of *mTORC1* [52, 53]. The research showed that mTOR inhibition could not extend longevity of ATG-deficient worms, suggesting that the process of modulating lifespan through the mTOR signaling pathway might depend on autophagy mechanisms [54]. In addition, the rapamycin was found to extend lifespan

in mice, further indicating the stimulative ageing effect of mTOR signaling pathway in the cellular senescence [55]. Based on the above studies, we believe that cellular senescence is a vital biological process in regulating LUAD via the mTOR signaling pathway. Furthermore, the JAK/STAT signaling pathway is also considered to be closely associated with biological behaviors of lung cancer, such as drug resistance [56]. Xu et al. verified that *JAK2* was positively linked to the proliferation and invasion of A549 cells [57]. There are also some evidences ~~indicate~~ that the JAK/STAT signaling pathway in senescent cells is more active than non-senescent cells and inhibition of the pathway can suppress the aging-related secretory phenotype and alleviate dysfunction in senescent cells [58].

The analysis of TIME showed StromalScore, ImmuneScore and ESTIMATEScore were significantly higher in low-risk groups. Therefore, we speculated that antitumor immunotherapy had a good therapeutic effect for the low-risk patients. Subsequently, ssGSEA also revealed a variety of immune cells, as well as some immune-related pathways, had higher immune scores in the group with low risk. Mayoux et al. demonstrated that DCs determined the treatment efficacy of PD-L1 immune checkpoint inhibitors [59]. A DC gene signature was strongly related to improvement of the OS in NSCLC patients through PD-L1 blockade treatment. ~~Qu et al. discovered that the release of CCL5 from A549 cells could promote migration of the human MC cells in vitro. Moreover, when a human MC cell line (HMC-1) and A549 were transplanted together in nude mice, MCs induced β-catenin phosphorylation accelerate growth of tumor through release of IL-8 [58].~~ Shikotra et al. discovered that

the mast cells with different phenotypes were increased in the NSCLC, and the production of TNF-α by both mast cell phenotypes was particularly important for their interaction with other immune cells and the associated inhibition of tumor progression [60]. Researchers found that the Tregs with increased number and enhanced activity in the peripheral blood, metastatic lymph nodes and the tumors were positively related to the recurrence and metastasis of patients with NSCLC [61, 62]. The immune checkpoint inhibitors were received more and more attention in recent years. We further analyzed the expression condition of genes related to immune checkpoint and revealed that the majority of checkpoints were more active in the low-risk group. These outcomes demonstrated that the patients at low risk were probably more responsive to immunotherapy, and the immune checkpoint inhibitors would improve the prognosis of these patients. Among these immune checkpoints, CTLA-4 inhibitors, as the effective drugs, were a milestone in the treatment of NSCLC [63]. Besides, *TIGIT* and *CD27* related immune checkpoints inhibitors have been under investigation in preclinical phase [64]. Lastly, our research showed that it was probably more advantageous for high-risk patients to accept the conventional chemotherapy drugs (cisplatin, docetaxel, etoposide, paclitaxel, and gemcitabine) and targeted therapy (erlotinib, crizotinib and afatinib).

To sum up, most of the results were helpful to explore a more precise and personalized therapeutic schedule for LUAD patients. Nevertheless, some limitations of our design are inevitable. Firstly, this study still requires an appropriate external dataset to validate the applicability of the CS-RLPS. Secondly, the molecular mechanism of

cellular senescence-related lncRNAs in LUAD need~~s~~ further experimental verification.

Conclusion

In summary, the CS-RLPS can be regarded as an independently prognostic indicator of LUAD patients. It is helpful to explore the underlying molecular mechanism of lncRNAs related to cellular senescence in LUAD. From the perspective of clinical guidance, the CS-RLPS provides relatively scientific treatment strategies for LUAD patients. However, future experiments will be necessary to confirm its validity once more.

Acknowledgments

We express gratitude to contributions from TCGA database and CellAge database.

Author Contributions

Conception and design: AL, XW, YL and JZ.

Data collection: AL, XW and YL.

Statistical analysis and interpretation of data: AL, XW, DY, YY and YL.

Preparation of the figures: AL, XW, HZ, GL and JZ. The first draft writing: AL, XW and LH.

Review and revise of the manuscript: YL and JZ.

References

1. Sung H, Ferlay J, Siegel RL, Laversanne M, Soerjomataram I, Jemal A, et al. Global Cancer Statistics 2020: GLOBOCAN Estimates of Incidence and Mortality Worldwide for 36 Cancers in 185 Countries. CA Cancer J Clin. 2021;71(3):209-49. http://doi.org/10.3322/caac.21660
2. Thai AA, Solomon BJ, Sequist LV, Gainor JF, Heist RS. Lung cancer. Lancet. 2021;398(10299):535-54. http://doi.org/10.1016/S0140-6736(21)00312-3
3. Hernandez-Segura A, Nehme J, Demaria M. Hallmarks of Cellular Senescence. Trends Cell Biol. 2018;28(6):436-53. http://doi.org/10.1016/j.tcb.2018.02.001
4. Demaria M, O'Leary MN, Chang J, Shao L, Liu S, Alimirah F, et al. Cellular Senescence Promotes Adverse Effects of Chemotherapy and Cancer Relapse. Cancer Discov. 2017;7(2):165-76. http://doi.org/10.1158/2159-8290.CD-16-0241
5. Lasry A, Ben-Neriah Y. Senescence-associated inflammatory responses: aging and cancer perspectives. Trends Immunol. 2015;36(4):217-28. http://doi.org/10.1016/j.it.2015.02.009
6. Coppé JP, Desprez PY, Krtolica A, Campisi J. The senescence-associated secretory phenotype: the dark side of tumor suppression. Annu Rev Pathol. 2010;5:99-118. http://doi.org/10.1146/annurev-pathol-121808-102144
7. Lopes-Paciencia S, Saint-Germain E, Rowell MC, Ruiz AF, Kalegari P, Ferbeyre G. The senescence-associated secretory phenotype and its regulation. Cytokine. 2019;117:15-22. http://doi.org/10.1016/j.cyto.2019.01.013
8. Xue W, Zender L, Miething C, Dickins RA, Hernando E, Krizhanovsky V, et al. Senescence and tumour clearance is triggered by p53 restoration in murine liver carcinomas. Nature.

2007;445(7128):656-60. http://doi.org/10.1038/nature05529

9. Chen YX, Wang CJ, Xiao DS, He BM, Li M, Yi XP, et al. eIF3a R803K mutation mediates chemotherapy resistance by inducing cellular senescence in small cell lung cancer. Pharmacol Res. 2021;174:105934. http://doi.org/10.1016/j.phrs.2021.105934
10. Eggert T, Wolter K, Ji J, Ma C, Yevsa T, Klotz S, et al. Distinct Functions of Senescence-Associated Immune Responses in Liver Tumor Surveillance and Tumor Progression. Cancer Cell. 2016;30(4):533-47. http://doi.org/10.1016/j.ccell.2016.09.003
11. Kim YH, Choi YW, Lee J, Soh EY, Kim JH, Park TJ. Senescent tumor cells lead the collective invasion in thyroid cancer. Nat Commun. 2017;8:15208. http://doi.org/10.1038/ncomms15208
12. Statello L, Guo CJ, Chen LL, Huarte M. Gene regulation by long non-coding RNAs and its biological functions. Nat Rev Mol Cell Biol. 2021;22(2):96-118. http://doi.org/10.1038/s41580-020-00315-9
13. Wang KC, Chang HY. Molecular mechanisms of long noncoding RNAs. Mol Cell. 2011;43(6):904-14. http://doi.org/10.1016/j.molcel.2011.08.018
14. Fernandes JCR, Acuna SM, Aoki JI, Floeter-Winter LM, Muxel SM. Long Non-Coding RNAs in the Regulation of Gene Expression: Physiology and Disease. NON-CODING RNA. 2019;5(171). http://doi.org/10.3390/ncrna5010017
15. Peng F, Wang R, Zhang Y, Zhao Z, Zhou W, Chang Z, et al. Differential expression analysis at the individual level reveals a lncRNA prognostic signature for lung adenocarcinoma. Mol Cancer. 2017;16(1):98. http://doi.org/10.1186/s12943-017-0666-z
16. Yu G, Wang LG, Han Y, He QY. clusterProfiler: an R package for comparing biological themes among gene clusters. Omics. 2012;16(5):284-7. http://doi.org/10.1089/omi.2011.0118

17. Walter W, Sánchez-Cabo F, Ricote M. GOplot: an R package for visually combining expression data with functional analysis. Bioinformatics. 2015;31(17):2912-4. http://doi.org/10.1093/bioinformatics/btv300
18. Tibshirani R. The lasso method for variable selection in the Cox model. Stat Med. 1997;16(4):385-95. http://doi.org/10.1002/(sici)1097-0258(19970228)16:4<385::aid-sim380>3.0.co;2-3
19. Friedman J, Hastie T, Tibshirani R. Regularization Paths for Generalized Linear Models via Coordinate Descent. J Stat Softw. 2010;33(1):1-22.
20. Subramanian A, Tamayo P, Mootha VK, Mukherjee S, Ebert BL, Gillette MA, et al. Gene set enrichment analysis: a knowledge-based approach for interpreting genome-wide expression profiles. Proc Natl Acad Sci U S A. 2005;102(43):15545-50. http://doi.org/10.1073/pnas.0506580102
21. Yoshihara K, Shahmoradgoli M, Martínez E, Vegesna R, Kim H, Torres-Garcia W, et al. Inferring tumour purity and stromal and immune cell admixture from expression data. Nat Commun. 2013;4:2612. http://doi.org/10.1038/ncomms3612
22. Rooney MS, Shukla SA, Wu CJ, Getz G, Hacohen N. Molecular and genetic properties of tumors associated with local immune cytolytic activity. Cell. 2015;160(1-2):48-61. http://doi.org/10.1016/j.cell.2014.12.033
23. Geeleher P, Cox N, Huang RS. pRRophetic: an R package for prediction of clinical chemotherapeutic response from tumor gene expression levels. Plos One. 2014;9(9):e107468. http://doi.org/10.1371/journal.pone.0107468
24. Allemani C, Matsuda T, Di Carlo V, Harewood R, Matz M, Nikšić M, et al. Global surveillance of trends in cancer survival 2000-14 (CONCORD-3): analysis of individual records for 37 513 025

patients diagnosed with one of 18 cancers from 322 population-based registries in 71 countries. Lancet. 2018;391(10125):1023-75. http://doi.org/10.1016/S0140-6736(17)33326-3

25. Kuilman T, Michaloglou C, Mooi WJ, Peeper DS. The essence of senescence. Genes Dev. 2010;24(22):2463-79. http://doi.org/10.1101/gad.1971610

26. Calcinotto A, Kohli J, Zagato E, Pellegrini L, Demaria M, Alimonti A. Cellular Senescence: Aging, Cancer, and Injury. Physiol Rev. 2019;99(2):1047-78. http://doi.org/10.1152/physrev.00020.2018

27. Weber JD, Taylor LJ, Roussel MF, Sherr CJ, Bar-Sagi D. Nucleolar Arf sequesters Mdm2 and activates p53. Nat Cell Biol. 1999;1(1):20-6. http://doi.org/10.1038/8991

28. Kamijo T, Zindy F, Roussel MF, Quelle DE, Downing JR, Ashmun RA, et al. Tumor suppression at the mouse INK4a locus mediated by the alternative reading frame product p19ARF. Cell. 1997;91(5):649-59. http://doi.org/10.1016/s0092-8674(00)80452-3

29. Liu W, Zhuang C, Huang T, Yang S, Zhang M, Lin B, et al. Loss of CDKN2A at chromosome 9 has a poor clinical prognosis and promotes lung cancer progression. Mol Genet Genomic Med. 2020;8(12):e1521. http://doi.org/10.1002/mgg3.1521

30. Li S, Li H, Cao Y, Geng H, Ren F, Li K, et al. Integrated bioinformatics analysis reveals CDK1 and PLK1 as potential therapeutic targets of lung adenocarcinoma. Medicine (Baltimore). 2021;100(32):e26474. http://doi.org/10.1097/MD.0000000000026474

31. Li M, He F, Zhang Z, Xiang Z, Hu D. CDK1 serves as a potential prognostic biomarker and target for lung cancer. J Int Med Res. 2020;48(2):1219697060. http://doi.org/10.1177/0300060519897508

32. Chen C, Tang Y, Qu WD, Han X, Zuo JB, Cai QY, et al. Evaluation of clinical value and potential mechanism of MTFR2 in lung adenocarcinoma via bioinformatics. Bmc Cancer. 2021;21(1):619. http://doi.org/10.1186/s12885-021-08378-3

33. Li Q, Bian Y, Li Q. Down-Regulation of TMPO-AS1 Induces Apoptosis in Lung Carcinoma Cells by Regulating miR-143-3p/CDK1 Axis. Technol Cancer Res Treat. 2021;20:1079216528. http://doi.org/10.1177/1533033820948880

34. Fischer M. Census and evaluation of p53 target genes. Oncogene. 2017;36(28):3943-56. http://doi.org/10.1038/onc.2016.502

35. Gu Z, Jiang J, Tan W, Xia Y, Cao H, Meng Y, et al. p53/p21 Pathway involved in mediating cellular senescence of bone marrow-derived mesenchymal stem cells from systemic lupus erythematosus patients. Clin Dev Immunol. 2013;2013:134243. http://doi.org/10.1155/2013/134243

36. Li H, Zhang H, Huang G, Bing Z, Xu D, Liu J, et al. Loss of RPS27a expression regulates the cell cycle, apoptosis, and proliferation via the RPL11-MDM2-p53 pathway in lung adenocarcinoma cells. J Exp Clin Cancer Res. 2022;41(1):33. http://doi.org/10.1186/s13046-021-02230-z

37. Shen D, Zeng Y, Zhang W, Li Y, Zhu J, Liu Z, et al. Chenodeoxycholic acid inhibits lung adenocarcinoma progression via the integrin α5β1/FAK/p53 signaling pathway. Eur J Pharmacol. 2022;923:174925. http://doi.org/10.1016/j.ejphar.2022.174925

38. Roth A, Boulay K, Groß M, Polycarpou-Schwarz M, Mallette FA, Regnier M, et al. Targeting LINC00673 expression triggers cellular senescence in lung cancer. Rna Biol. 2018;15(12):1499-511. http://doi.org/10.1080/15476286.2018.1553481

39. Tao Y, Yue P, Miao Y, Gao S, Wang B, Leng SX, et al. The lncRNA MEG3/miR-16-5p/VGLL4 regulatory axis is involved in etoposide-induced senescence of tumor cells. J Gene Med. 2021;23(2):e3291. http://doi.org/10.1002/jgm.3291

40. Li L, Huang Y, Gao Y, Shi T, Xu Y, Li H, et al. EGF/EGFR upregulates and cooperates with Netrin-4 to protect glioblastoma cells from DNA damage-induced senescence. Bmc Cancer.

2018;18(1):1215. http://doi.org/10.1186/s12885-018-5056-4

41. Tam CW, Liu VW, Leung WY, Yao KM, Shiu SY. The autocrine human secreted PDZ domain-containing protein 2 (sPDZD2) induces senescence or quiescence of prostate, breast and liver cancer cells via transcriptional activation of p53. Cancer Lett. 2008;271(1):64-80. http://doi.org/10.1016/j.canlet.2008.05.047

42. Phadke M, Krynetskaia N, Mishra A, Krynetskiy E. Accelerated cellular senescence phenotype of GAPDH-depleted human lung carcinoma cells. Biochem Biophys Res Commun. 2011;411(2):409-15. http://doi.org/10.1016/j.bbrc.2011.06.165

43. Sun M, Sheng H, Wu T, Song J, Sun H, Wang Y, et al. PIKE-A promotes glioblastoma growth by driving PPP flux through increasing G6PD expression mediated by phosphorylation of STAT3. Biochem Pharmacol. 2021;192:114736. http://doi.org/10.1016/j.bcp.2021.114736

44. Li J, Liu R, Sun M, Wang J, Wang N, Zhang X, et al. The FcεRI signaling pathway is involved in the pathogenesis of lacrimal gland benign lymphoepithelial lesions as shown by transcriptomic analysis. Sci Rep. 2021;11(1):21853. http://doi.org/10.1038/s41598-021-01395-z

45. Wang Q, Liu Z, Du K, Liang M, Zhu X, Yu Z, et al. Babaodan inhibits cell growth by inducing autophagy through the PI3K/AKT/mTOR pathway and enhances antitumor effects of cisplatin in NSCLC cells. Am J Transl Res. 2019;11(8):5272-83.

46. Hu T, Shen H, Huang H, Yang Z, Zhou Y, Zhao G. Cholesterol-lowering drug pitavastatin targets lung cancer and angiogenesis via suppressing prenylation-dependent Ras/Raf/MEK and PI3K/Akt/mTOR signaling. Anticancer Drugs. 2020;31(4):377-84. http://doi.org/10.1097/CAD.0000000000000885

47. Chen M, Zhu LL, Su JL, Li GL, Wang J, Zhang YN. Prucalopride inhibits lung cancer cell

proliferation, invasion, and migration through blocking of the PI3K/AKT/mTor signaling pathway. Hum Exp Toxicol. 2020;39(2):173-81. http://doi.org/10.1177/0960327119883409

48. Waqar SN, Gopalan PK, Williams K, Devarakonda S, Govindan R. A phase I trial of sunitinib and rapamycin in patients with advanced non-small cell lung cancer. Chemotherapy. 2013;59(1):8-13. http://doi.org/10.1159/000348584

49. Waqar SN, Baggstrom MQ, Morgensztern D, Williams K, Rigden C, Govindan R. A Phase I Trial of Temsirolimus and Pemetrexed in Patients with Advanced Non-Small Cell Lung Cancer. Chemotherapy. 2016;61(3):144-7. http://doi.org/10.1159/000442147

50. Liu GY, Sabatini DM. mTOR at the nexus of nutrition, growth, ageing and disease. Nat Rev Mol Cell Biol. 2020;21(4):183-203. http://doi.org/10.1038/s41580-019-0199-y

51. Carroll B, Nelson G, Rabanal-Ruiz Y, Kucheryavenko O, Dunhill-Turner NA, Chesterman CC, et al. Persistent mTORC1 signaling in cell senescence results from defects in amino acid and growth factor sensing. J Cell Biol. 2017;216(7):1949-57. http://doi.org/10.1083/jcb.201610113

52. Laberge RM, Sun Y, Orjalo AV, Patil CK, Freund A, Zhou L, et al. MTOR regulates the pro-tumorigenic senescence-associated secretory phenotype by promoting IL1A translation. Nat Cell Biol. 2015;17(8):1049-61. http://doi.org/10.1038/ncb3195

53. Herranz N, Gallage S, Mellone M, Wuestefeld T, Klotz S, Hanley CJ, et al. mTOR regulates MAPKAPK2 translation to control the senescence-associated secretory phenotype. Nat Cell Biol. 2015;17(9):1205-17. http://doi.org/10.1038/ncb3225

54. Tóth ML, Sigmond T, Borsos E, Barna J, Erdélyi P, Takács-Vellai K, et al. Longevity pathways converge on autophagy genes to regulate life span in Caenorhabditis elegans. Autophagy. 2008;4(3):330-8. http://doi.org/10.4161/auto.5618

55. Arriola AS, Pumper CP, Baar EL, Cummings NE, Lamming DW. Intermittent Administration of Rapamycin Extends the Life Span of Female C57BL/6J Mice. J Gerontol A Biol Sci Med Sci. 2016;71(7):876-81. http://doi.org/10.1093/gerona/glw064

56. Li SD, Ma M, Li H, Waluszko A, Sidorenko T, Schadt EE, et al. Cancer gene profiling in non-small cell lung cancers reveals activating mutations in JAK2 and JAK3 with therapeutic implications. Genome Med. 2017;9(1):89. http://doi.org/10.1186/s13073-017-0478-1

57. Xu Y, Jin J, Xu J, Shao YW, Fan Y. JAK2 variations and functions in lung adenocarcinoma. Tumour Biol. 2017;39(6):1393378524. http://doi.org/10.1177/1010428317711140

58. Xu M, Tchkonia T, Ding H, Ogrodnik M, Lubbers ER, Pirtskhalava T, et al. JAK inhibition alleviates the cellular senescence-associated secretory phenotype and frailty in old age. Proc Natl Acad Sci U S A. 2015;112(46):E6301-10. http://doi.org/10.1073/pnas.1515386112

59. Mayoux M, Roller A, Pulko V, Sammicheli S, Chen S, Sum E, et al. Dendritic cells dictate responses to PD-L1 blockade cancer immunotherapy. Sci Transl Med. 2020;12(534). http://doi.org/10.1126/scitranslmed.aav7431

60. Shikotra A, Ohri CM, Green RH, Waller DA, Bradding P. Mast cell phenotype, TNFα expression and degranulation status in non-small cell lung cancer. Sci Rep. 2016;6:38352. http://doi.org/10.1038/srep38352

61. Erfani N, Mehrabadi SM, Ghayumi MA, Haghshenas MR, Mojtahedi Z, Ghaderi A, et al. Increase of regulatory T cells in metastatic stage and CTLA-4 over expression in lymphocytes of patients with non-small cell lung cancer (NSCLC). Lung Cancer. 2012;77(2):306-11. http://doi.org/10.1016/j.lungcan.2012.04.011

62. Schneider T, Kimpfler S, Warth A, Schnabel PA, Dienemann H, Schadendorf D, et al. Foxp3(+)

regulatory T cells and natural killer cells distinctly infiltrate primary tumors and draining lymph nodes in pulmonary adenocarcinoma. J Thorac Oncol. 2011;6(3):432-8. http://doi.org/10.1097/JTO.0b013e31820b80ca

63. Paz-Ares L, Ciuleanu TE, Cobo M, Schenker M, Zurawski B, Menezes J, et al. First-line nivolumab plus ipilimumab combined with two cycles of chemotherapy in patients with non-small-cell lung cancer (CheckMate 9LA): an international, randomised, open-label, phase 3 trial. Lancet Oncol. 2021;22(2):198-211. http://doi.org/10.1016/S1470-2045(20)30641-0

64. Bewersdorf JP, Shallis RM, Zeidan AM. Immune checkpoint inhibition in myeloid malignancies: Moving beyond the PD-1/PD-L1 and CTLA-4 pathways. Blood Rev. 2021;45:100709. http://doi.org/10.1016/j.blre.2020.100709

Supporting information

S1 Fig. A forest plot for 66 lncRNAs related to prognosis of LUAD.

S1 Table. ~~Clinicopathological characteristics of LUAD patitents from TCGA.~~ 279 cellular senescence-related genes.

S2 Table. 62 cellular senescence-related DEGs.

S3 Table. 1081 cellular senescence-related lncRNAs.

S4 Table. Clinicopathological characteristics of LUAD patitents from TCGA.

S5 Table. 66 lncRNAs connected to the prognosis of LUAD.

S6 Table. 29 cellular senescence-related lncRNAs by LASSO.

S7 Table. 6 cellular senescence-related lncRNAs by multivariate Cox regression

analysis.

S1 File. 6 lncRNAs recheck and calculations of coding potential.

Dear Editor:

Thank you for giving us the opportunity to revise our manuscript entitled “A novel cellular senescence-related lncRNAs predictive signature for the prognosis of lung adenocarcinoma”. We appreciate the reviewers’ constructive comments and their enthusiasm for our findings. Our point-to-point response to the issues raised by the referees is included. I hope that you would find out the revision is substantial and the reviewers’ concerns have been adequately addressed and agree that the manuscript is now acceptable for publication in PLOS ONE.

Sincerely,

Yun Li
liyun28@mail.sysu.edu.cn
Department of Thoracic Surgery, The Seventh Affiliated Hospital, Sun Yat-sen University, Shenzhen, China

Response to Editor’s comments

1. Please ensure that your manuscript meets PLOS ONE's style requirements, including those for file naming.

Authors: Thanks for your reminding. We have rechecked our manuscript and introduced minor changes to comply with the journal’s style requirements.

2. Please note that PLOS ONE has specific guidelines on code sharing for submissions in which author-generated code underpins the findings in the manuscript. In these cases, all author-generated code must be made available without restrictions upon publication of the work.

Authors: We are very happy to share our code if necessary.

3. About the financial disclosure.

Authors: The authors received no specific funding for this work.

4. In your Data Availability statement, you have not specified where the minimal data set underlying the results described in your manuscript can be found. PLOS defines a study's minimal data set as the underlying data used to reach the conclusions drawn in the manuscript and any additional data required to replicate the reported study findings in their entirety. All PLOS journals require that the minimal data set be made fully available.

Authors: According to your requirements, we will upload our study’s minimal underlying data set as supporting information when we re-submit our revised manuscript.

5. PLOS requires an ORCID iD for the corresponding author in Editorial Manager on papers submitted after December 6th, 2016. Please ensure that you have an ORCID iD and that it is validated in Editorial Manager. To do this, go to ‘Update my Information’ (in the upper left-hand corner of the main menu), and click on the Fetch/Validate link next to the ORCID field. This will take you to the ORCID site and

allow you to create a new iD or authenticate a pre-existing iD in Editorial Manager.
Authors: We have already had an ORCID iD and validated it in Editorial Manager. The ORCID iD of the corresponding author is 0000-0003-0949-6519.

6. About the references.
Authors: We appreciate greatly your important warnings and recheck our references. The 14th reference may involve academic misconduct. The 58th reference have been retracted, so we removed the two references and replaced them with relevant current references. Meanwhile, we added two references （the 16th, 17th）and revised the original contents accordingly.

Response to Reviewers' comments
Reviewer #1:
1. Did the authors review and double check the fidelity of the lncRNAs from the databases such as NONCODE etc? For example, C20orf ids indeed might have been reannotated and they could have been coding chunks by the time the paper came out.
Authors: We have rechecked and ensured the fidelity of the 6 lncRNAs in signature from the LNCipedia database. We found the new annotation for the 6 lncRNAs and searched the relevant literature. For example, the alternative gene name of C20orf197 is lnc-CDH26-9, or LINC02910. The following paper came out also mentions the C20orf197. The others' annotation and relevant paper was shown in our uploaded **S1 File**.
Yao J, Chen X, Liu X, Li R, Zhou X, Qu Y. Characterization of a ferroptosis and iron-metabolism related lncRNA signature in lung adenocarcinoma. Cancer Cell Int. 2021;21(1):340. http://doi.org/10.1186/s12935-021-02027-2

2. Are there any coding potential calculations that the authors looked into these lncRNAs esp looking them as guides (cis or trans)?
Authors: We analyzed the coding potential of the 6 lncRNAs based on the LNCipedia database. All lncRNAs were determined to have no coding ability. The outcome of coding potential is shown in our uploaded **S1 File**.

3. The PPI and KEGG is well taken, but it would have been nice had authors shown the lncRNA-protein interactions as well to check regulation.
Authors: We showed the correlation analysis between the 6 lncRNAs and coding RNAs in **Fig 3C**. Regretfully, the interaction mechanism between lncRNAs and protein is so complicated that we are difficult to visualize them.

4. The abstract must be rewritten.
Authors: We reviewed the section carefully and recognize the seriousness of the problem. We have rewritten and improved the overall structure and logicality of the abstract. The revised abstract will meet hopefully your requirements.

5. The figures are of not high resolution, may be inserted with HR images.
Authors: Thanks for your precious advice. We have revised and improved the resolution of all the figures.

6. Minor but essential, please correct the following:
L61: hepatocellular carcinoma is CLOSE to the WORST overall survival
L192: 39 days OLD
L198: lncRNAs WERE
L201; was SHOWN
l237: scatter plot (tw different words ?)
L389 and L417: through THE
L431: risk groupS
L449: improve THE prognosis
L461: need (remove s)
Authors: We apologize for the mistakes of our manuscript and have revised the above mistakes one by one. We will be happy to edit the text further, based on helpful comments from the reviewers.

Reviewer #2:
1. The study is good. However, needs improvements in terms of overall English language in sections of Abstract, Methods and Results. Please invest enough time and a revision with main authors to bring out the best of the analysis in the text.
Authors: We apologize for the poor language of our manuscript. We have worked on the manuscript for a long time and carefully revised the content of Abstract, Methods and Result, including grammar, conciseness, readability, and logicality. Deletions and additions are both marked. We hope that the language level has been substantially improved.

2. An alternative title could be: "Predictive molecular signature consisting of lncRNA related to cellular senescence for the prognosis of lung adenocarcinoma ". This would be more appropriate.
Authors: That’s a good idea. Thanks for your considerate suggestion. We think that the alternative title is more in line with the overall content of this manuscript. We decide to replace the original title with the new one. Moreover, we would like ask for your opinion. Whether we need add an “A” at the beginning of the new title. “A predictive molecular signature consisting of lncRNAs related to cellular senescence for the prognosis of lung adenocarcinoma”.

3. Materials and methods section could start with explaining the flow diagram of analysis.
Authors: We have adjusted the writing order of the Materials and methods according to the sequence of the flow diagram. It is very sorry that we did not fully understand your advice. Whether you require us to add an explanation of the flow diagram at the

beginning of the Materials and methods section or not? Please let us know if it's necessary and we would like to follow up your suggestion.

4. Results section – Each subtitle in this section should highlight the finding or outcome of analysis rather than the process or type of analysis! Please revise, this is very important to improve the quality of presentation of results and also to reach the quality of journal.
Authors: We appreciate greatly your pointing out the shortcomings and have already recognized the seriousness of the problem. We have carefully revised each subtitle to highlight the finding or outcome of analysis.

5. Figures: Please try to improve the resolution of all the figures. Texts in some of the figures is not readable at all.
Authors: We are so sorry to bring poor visual experience to you. We have revised and improved the resolution of all the figures.
