# Supplementary material for: A predictive molecular signature consisting of lncRNAs associated with cellular senescence for the prognosis of lung adenocarcinoma
Source: PLoS One. 2023 Jun 23;18(6):e0287132. doi: 10.1371/journal.pone.0287132 (PMC10289466; doi:10.1371/journal.pone.0287132)
Supplement: Attachment — Submitted filename: Response to Reviewers.docx [file pone.0287132.s010.docx]

Dear Editor:

Thank you for giving us the opportunity to revise our manuscript entitled “A novel cellular senescence-related lncRNAs predictive signature for the prognosis of lung adenocarcinoma”. We appreciate the reviewers’ constructive comments and their enthusiasm for our findings. Our point-to-point response to the issues raised by the referees is included. I hope that you would find out the revision is substantial and the reviewers’ concerns have been adequately addressed and agree that the manuscript is now acceptable for publication in PLOS ONE.

Sincerely,

Yun Li

[liyun28@mail.sysu.edu.cn](mailto:liyun28@mail.sysu.edu.cn)

Department of Thoracic Surgery, The Seventh Affiliated Hospital, Sun Yat-sen University, Shenzhen, China

Response to Editor’s comments

1. Please ensure that your manuscript meets PLOS ONE's style requirements, including those for file naming.

**Authors:** Thanks for your reminding. We have rechecked our manuscript and introduced minor changes to comply with the journal’s style requirements.

2. Please note that PLOS ONE has specific guidelines on code sharing for submissions in which author-generated code underpins the findings in the manuscript. In these cases, all author-generated code must be made available without restrictions upon publication of the work.

**Authors:** We are very happy to share our code if necessary.

3. About the financial disclosure.

**Authors:** The authors received no specific funding for this work.

4. In your Data Availability statement, you have not specified where the minimal data set underlying the results described in your manuscript can be found. PLOS defines a study's minimal data set as the underlying data used to reach the conclusions drawn in the manuscript and any additional data required to replicate the reported study findings in their entirety. All PLOS journals require that the minimal data set be made fully available.

**Authors:** According to your requirements, we will upload our study’s minimal underlying data set as supporting information when we re-submit our revised manuscript.

5. PLOS requires an ORCID iD for the corresponding author in Editorial Manager on papers submitted after December 6th, 2016. Please ensure that you have an ORCID iD and that it is validated in Editorial Manager. To do this, go to ‘Update my Information’ (in the upper left-hand corner of the main menu), and click on the Fetch/Validate link next to the ORCID field. This will take you to the ORCID site and allow you to create a new iD or authenticate a pre-existing iD in Editorial Manager.

Authors: We have already had an ORCID iD and validated it in Editorial Manager. The ORCID iD of the corresponding author is 0000-0003-0949-6519.

6. About the references.

**Authors:** We appreciate greatly your important warnings and recheck our references. The 14th reference may involve academic misconduct. The 58th reference have been retracted, so we removed the two references and replaced them with relevant current references. Meanwhile, we added two references （the 16th, 17th）and revised the original contents accordingly.

Response to Reviewers’ comments

Reviewer #1:

1. Did the authors review and double check the fidelity of the lncRNAs from the databases such as NONCODE etc? For example, C20orf ids indeed might have been reannotated and they could have been coding chunks by the time the paper came out.

**Authors:** We have rechecked and ensured the fidelity of the 6 lncRNAs in signature from the LNCipedia database. We found the new annotation for the 6 lncRNAs and searched the relevant literature. For example, the alternative gene name of C20orf197 is lnc-CDH26-9, or LINC02910. The following paper came out also mentions the C20orf197. The others’ annotation and relevant paper was shown in our uploaded **S1 File**.

Yao J, Chen X, Liu X, Li R, Zhou X, Qu Y. Characterization of a ferroptosis and iron-metabolism related lncRNA signature in lung adenocarcinoma. Cancer Cell Int. 2021;21(1):340. <http://doi.org/10.1186/s12935-021-02027-2>

2. Are there any coding potential calculations that the authors looked into these lncRNAs esp looking them as guides (cis or trans)?

**Authors:** We analyzed the coding potential of the 6 lncRNAs based on the LNCipedia database. All lncRNAs were determined to have no coding ability. The outcome of coding potential is shown in our uploaded **S1 File**.

3. The PPI and KEGG is well taken, but it would have been nice had authors shown the lncRNA-protein interactions as well to check regulation.

**Authors:** We showed the correlation analysis between the 6 lncRNAs and coding RNAs in **Fig 3C**. Regretfully, the interaction mechanism between lncRNAs and protein is so complicated that we are difficult to visualize them.

4. The abstract must be rewritten.

**Authors:** We reviewed the section carefully and recognize the seriousness of the problem. We have rewritten and improved the overall structure and logicality of the abstract. The revised abstract will meet hopefully your requirements.

5. The figures are of not high resolution, may be inserted with HR images.

**Authors:** Thanks for your precious advice. We have revised and improved the resolution of all the figures.

6. Minor but essential, please correct the following:
   L61: hepatocellular carcinoma is CLOSE to the WORST overall survival
   L192: 39 days OLD
   L198: lncRNAs WERE
   L201; was SHOWN
   l237: scatter plot (tw different words ?)
   L389 and L417: through THE
   L431: risk groupS
   L449: improve THE prognosis
   L461: need ( remove s)

**Authors:** We apologize for the mistakes of our manuscript and have revised the above mistakes one by one. We will be happy to edit the text further, based on helpful comments from the reviewers.

Reviewer #2:

1. The study is good. However, needs improvements in terms of overall English language in sections of Abstract, Methods and Results. Please invest enough time and a revision with main authors to bring out the best of the analysis in the text.

**Authors:** We apologize for the poor language of our manuscript. We have worked on the manuscript for a long time and carefully revised the content of Abstract, Methods and Result, including grammar, conciseness, readability, and logicality. Deletions and additions are both marked. We hope that the language level has been substantially improved.

2. An alternative title could be: "Predictive molecular signature consisting of lncRNA related to cellular senescence for the prognosis of lung adenocarcinoma ". This would be more appropriate.

**Authors:** That’s a good idea. Thanks for your considerate suggestion. We think that the alternative title is more in line with the overall content of this manuscript. We decide to replace the original title with the new one. Moreover, we would like ask for your opinion. Whether we need add an “A” at the beginning of the new title. “A predictive molecular signature consisting of lncRNAs related to cellular senescence for the prognosis of lung adenocarcinoma”.

3. Materials and methods section could start with explaining the flow diagram of analysis.

Authors: We have adjusted the writing order of the Materials and methods according to the sequence of the flow diagram. It is very sorry that we did not fully understand your advice. Whether you require us to add an explanation of the flow diagram at the beginning of the Materials and methods section or not? Please let us know if it's necessary and we would like to follow up your suggestion.

4. Results section – Each subtitle in this section should highlight the finding or outcome of analysis rather than the process or type of analysis! Please revise, this is very important to improve the quality of presentation of results and also to reach the quality of journal.

Authors: We appreciate greatly your pointing out the shortcomings and have already recognized the seriousness of the problem. We have carefully revised each subtitle to highlight the finding or outcome of analysis.

5. Figures: Please try to improve the resolution of all the figures. Texts in some of the figures is not readable at all.

Authors: We are so sorry to bring poor visual experience to you. We have revised and improved the resolution of all the figures.
