# Supplementary material for: A predictive molecular signature consisting of lncRNAs associated with cellular senescence for the prognosis of lung adenocarcinoma
Source: PLoS One. 2023 Jun 23;18(6):e0287132. doi: 10.1371/journal.pone.0287132 (PMC10289466; doi:10.1371/journal.pone.0287132)
Supplement: Attachment — Submitted filename: Response to Reviewers.docx [file pone.0287132.s012.docx]

Dear Editor:

Thank you for giving us the opportunity to revise our manuscript entitled “A predictive molecular signature consisting of lncRNAs associated with cellular senescence for the prognosis of lung adenocarcinoma”. We appreciate the referee’s constructive comments and their enthusiasm for our findings. Our point-to-point response to the issues raised by the referees is included. I hope that you would find out the revision is substantial and the referees’ concerns have been adequately addressed and agree that the manuscript is now acceptable for publication in PLOS ONE.

Sincerely,

Yun Li

[liyun28@mail.sysu.edu.cn](mailto:Liyun28@mail.sysu.edu.cn)

Department of Thoracic Surgery, The Seventh Affiliated Hospital, Sun Yat-sen University, Shenzhen, China

Response to Reviewers’ comments

Reviewer #1:

1. I am satisfied with all the changes rendered. Thank you. Pl double check the language for brevity. The figure smay be of HR.

Authors: Thanks for your review. We have checked the language and made much revision again to ensure its brevity and correctness. Meanwhile, the resolution of all the figures has been improved.

Reviewer #2:

1. Similar article has appeared in Frontiers Genetics2022:
   A novel senescence-related lncRNA signature that predicts prognosis and the tumor microenvironment in patients with lung adenocarcinoma.
   Fang X, Huang E, Xie X, Yang K, Wang S, Huang X, Song M. Front Genet. 2022 Nov 3; 13:951311. doi: 10.3389/fgene.2022.951311. eCollection 2022.
   Please check it and if it’s needs to be quoted and if it has any of the lncRNA identified by your study are also outcome of this study, which might strengthen your claims. One is seen - AC026355.1.

Authors: That’s a good idea. We think that the article is valuable and we have quoted it as the 40th reference.

[38]	Fang X, Huang E, Xie X, Yang K, Wang S, Huang X, et al. A novel senescence-related lncRNA signature that predicts prognosis and the tumor microenvironment in patients with lung adenocarcinoma. Front Genet. 2022; 13:951311. http://doi.org/10.3389/fgene.2022.951311

2. Abstract – line one, and there is still scope to improve the abstract furthermore with language.
   # line 239-240 –pathogenesis and clinical factors.
   #447 - “Independently” -> independent.

Authors: Thanks for your point. We think that the “clinicopathological” is appropriate, because we found the usage in the following article.

*Chen M, Nie Z, Li Y, Gao Y, Wen X, Cao H, et al. A New Ferroptosis-Related lncRNA Signature Predicts the Prognosis of Bladder Cancer Patients. Front Cell Dev Biol. 2021; 9:699804. http://doi.org/10.3389/fcell.2021.699804*

According to your comments, we have addressed the rest of problems.

3. If all the lncRNA that are found in this study are already shown (demonstrated) in other studies functionally - that they are involved in cellular senescence, then only it would be appropriate to quote these lnRNAs as “cellular-senescence-related” otherwise it would be more appropriate to quote them as “Cellular senescence-associated-lncRNA”, and in entire text.

Authors: We think that raised point is precise. All the “cellular senescence-related lncRNAs” have been replaced with “Cellular senescence-associated lncRNA”.

4. line 67-68 - it's contrary to reports stating that - cellular senescence hinders or delays tumorigenesis!! Cellular senescence is a mechanism by which organism delays the onset of tumorigenesis.

Authors: We appreciate greatly you for pointing out the shortcomings and have recognized the seriousness of the problem. We have carefully revised it and changed the 3rd and 4th references.

[3]	Lee S, Lee JS. Cellular senescence: a promising strategy for cancer therapy. Bmb Rep. 2019;52(1):35-41. http://doi.org/10.5483/BMBRep.2019.52.1.294

[4]	Demirci D, Dayanc B, Mazi FA, Senturk S. The Jekyll and Hyde of Cellular Senescence in Cancer. Cells-Basel. 2021;10(2). http://doi.org/10.3390/cells10020208

5. A) Line 247-249 -“Risk score = (-0.270×AC026355.1 expression, 0.231×AL365181.2 expression, -0.616×AF131215.5 expression, -0.580×C20orf197 expression, -0.627×GAS6-AS1 expression, 0.505×GSEC expression)”.
   # in this formula: What is meant by ","(a comma)? Does it mean addition (+) ? And ‘–’ Does this hyphen mean “minus/subtraction”?

Authors: We are so sorry for this mistake. ","(a comma) means addition (+). ‘–’ means negative correlation coefficient.

Risk score = (-0.270×*AC026355.1* expression) + (0.231×*AL365181.2* expression) + (-0.616×*AF131215.5* expression) + (-0.580×*C20orf197* expression) + (-0.627×*GAS6-AS1* expression) + (0.505×*GSEC* expression).

B) Please check in the text attached pdf file with marking and comment.224 / 257 /295 -296 / 330 / 346-347 and at some other points. #414 - Therefore the enrichment result suggests that the… # 468 – indicating.

Authors: According to your comments, we have revised these issues one by one.

C) Result subtitles – It would be great if the subtitles would also contain or highlight the biological outcomes (in one line- or in a concise statement) explained under these subtitles.

Authors: Thanks for your suggestions. We have revised these subtitles to highlight the biological outcomes.
